# Carbene Radicals in Transition-Metal-Catalyzed Reactions

**DOI:** 10.1021/acscatal.3c00591

**Published:** 2023-04-06

**Authors:** Roel F.J. Epping, David Vesseur, Minghui Zhou, Bas de Bruin

**Affiliations:** † Homogeneous, Supramolecular and Bio-Inspired Catalysis Group, van ‘t Hoff Institute for Molecular Sciences (HIMS), University of Amsterdam, Science Park 904, 1098 XH Amsterdam, The Netherlands

**Keywords:** carbene radical, metallocarbene, transition metal catalysis, radical chemistry, metalloradical catalysis, cobalt catalysis

## Abstract

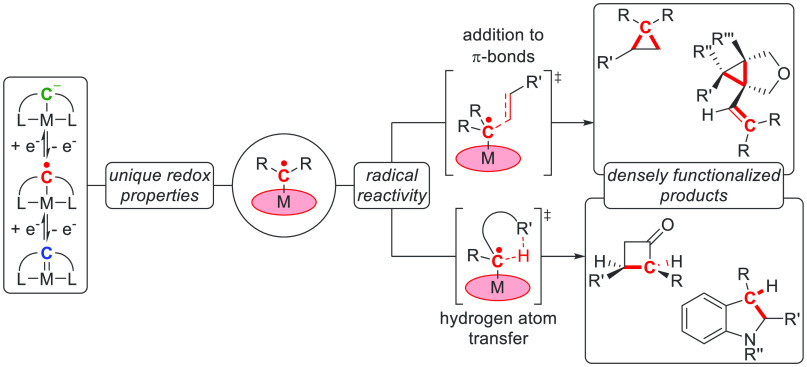

Discovered as organometallic curiosities in the 1970s, carbene radicals have become a staple in modern-day homogeneous catalysis. Carbene radicals exhibit nucleophilic radical-type reactivity orthogonal to classical electrophilic diamagnetic Fischer carbenes. Their successful catalytic application has led to the synthesis of a myriad of carbo- and heterocycles, ranging from simple cyclopropanes to more challenging eight-membered rings. The field has matured to employ densely functionalized chiral porphyrin-based platforms that exhibit high enantio-, regio-, and stereoselectivity. Thus far the focus has largely been on cobalt-based systems, but interest has been growing for the past few years to expand the application of carbene radicals to other transition metals. This Perspective covers the advances made since 2011 and gives an overview on the coordination chemistry, reactivity, and catalytic application of carbene radical species using transition metal complexes and catalysts.

## Introduction

Transition metal carbene chemistry has matured over the past few decades into an established field within both inorganic chemistry and homogeneous catalysis.^1^ Their applications range from unique electronic, magnetic, and photophysical properties to a wide variety of synthetic methods, such as several C–H and X–H insertion reactions,^2^ ring-forming and expansion transformations,^3^ alkene metathesis,^4^ and C1 polymerization^5^*inter alia*.

**Figure 1:**
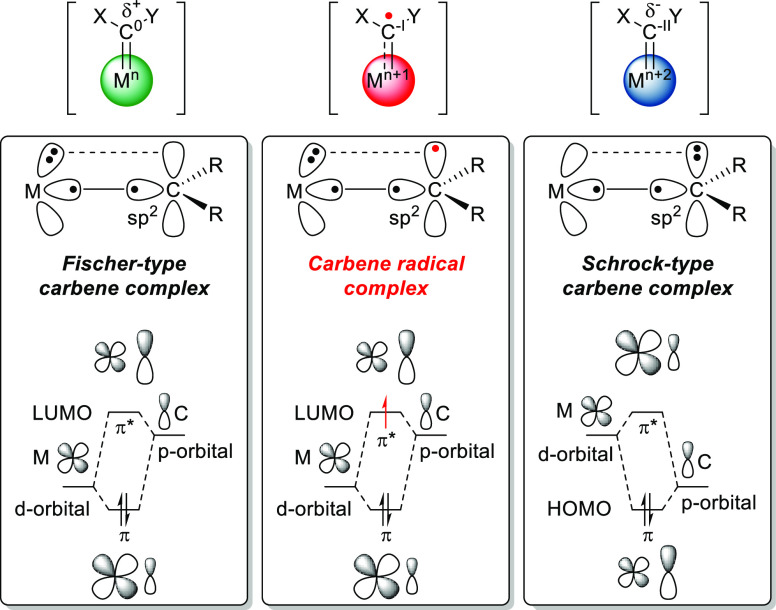
Schematic view of different electronic descriptors for metal carbenes (X/Y = neutral substituent) (top). Molecular orbital bonding scheme depicting the differences between Fischer-type and Schrock-type transition metal carbenes and the more recently established carbene radical (i.e., one-electron reduced Fischer-type carbene) (bottom).

**Scheme 1:**
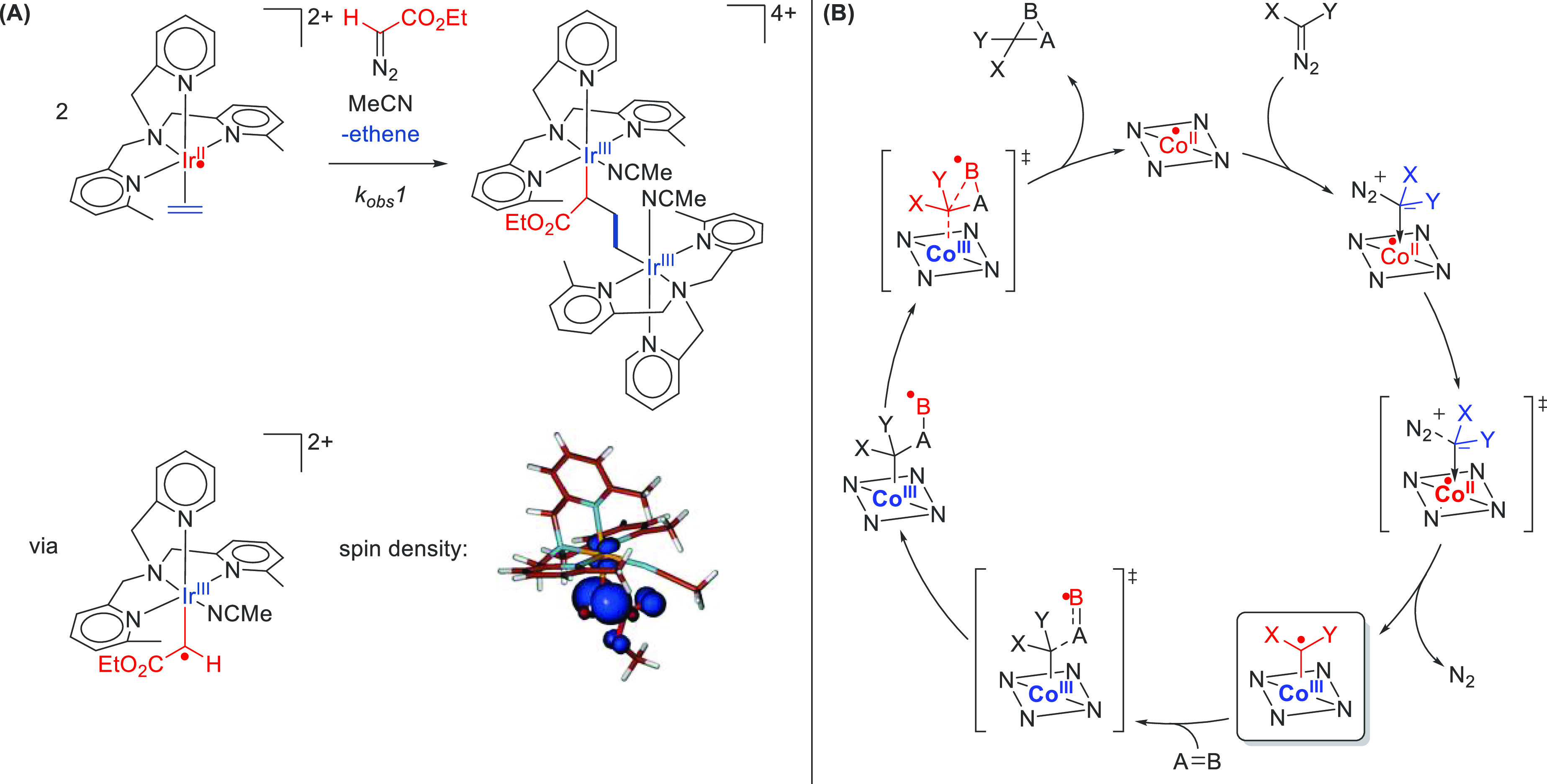
Reaction of [(Me_3_TPA)Ir^II^(Ethene)]^2+^ with Ethyl Diazoacetate Leading to Ethene–Carbene Coupling (A) The reaction proceeds via an Ir^III^–carbene radical intermediate. (B) General catalytic cycle involving the addition of double bonds to a diazo-derived cobalt(III)–carbene radical.

This rich and versatile organometallic class of compounds can be further divided into several types of metal carbenes depending on their electronic properties. Conventionally, two main types of carbenes exist: Fischer- and Schrock-type carbenes.^6^ Fischer-type carbenes generally feature metals in low oxidation states, mostly middle to late transition metals. Consequently, the metal–carbene π-bond of these systems is polarized toward the metal, resulting in a metal-centered π-HOMO and a carbon-centered π*-LUMO (Figure 1). Conversely, Schrock-type carbenes generally feature early transition metals in high oxidation states, and the metal–carbene π-bond of these systems is polarized toward the carbene carbon atom.^6b^ This results in a carbon-centered π-HOMO and a metal-centered π*-LUMO (Figure 1). Reactivity wise, the two are polar opposites; whereas Fischer-type carbenes are electrophilic on carbon, Schrock-type carbenes are nucleophilic. As summarized by Dzik et al.,^7^ the single-electron reduction of Fischer-type carbenes was first reported in the 1970s, leading to a third unique type of carbene, the carbene radical (Figure 1).^8^ Featuring a single electron in the carbon-centered SOMO (formerly the LUMO), these carbene radicals exhibit radical stepwise reactivity as opposed to the conventional two-electron concerted reactivity of Fischer-type carbenes. Moreover, they react in a nucleophilic fashion due to their reduced nature in contrast with the electrophilic nature of the parent Fischer-type carbene. Expanding on this initial reactivity, studies of group 9 metals in their 2+ oxidation states (Co, Rh, and Ir) featuring N-donor ligands revealed that these metals can generate carbene radical species in the absence of an external redox agent.^7,9^ Specifically, Co(II) complexes have been shown to be highly suitable for catalytic carbene transfer, operating via distinct single-electron stepwise radical-type pathways (Scheme 1B).^10^ These seminal discoveries have uncovered a new era of carbene chemistry, and significant advances have been made since then. Several reviews have covered certain aspects of the more recent discoveries. This Perspective gives an overview of the rich coordination chemistry and catalysis that transition metal carbene radicals have offered, expanding on the 2011 review by Dzik et al.^7^

## Coordination Chemistry and Reactivity

**Figure 2:**
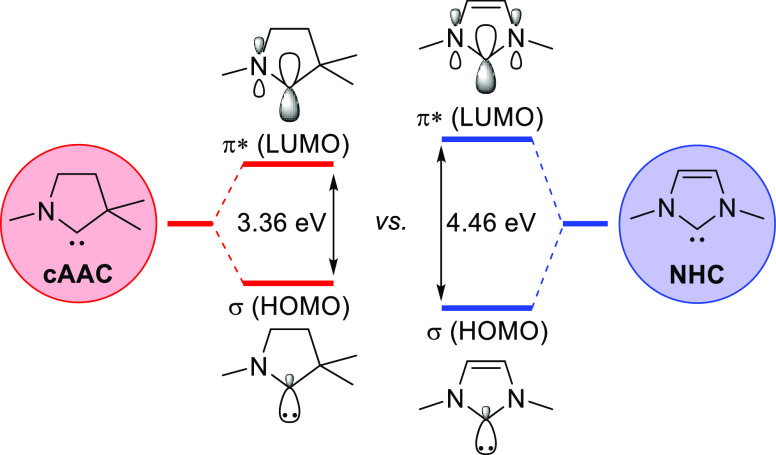
Frontier orbitals of a cyclic alkyl amino carbene (left, red) versus a classical *N*-heterocyclic carbene (right, blue). The decrease in π-donation leads to a smaller HOMO–LUMO gap for the cAAC ligand, giving access to noninnocent behavior.

Although well-known for their active role in carbene transfer, carbenes are also highly popular as stable organometallic ligands. The most well-known example of this is the family of *N*-heterocyclic Arduengo-type carbenes (NHCs), which are ubiquitous in modern organometallic chemistry and catalysis.^11^ The archetypical NHC is often derived from the aromatic imidazole heterocycle, leading to large HOMO–LUMO gaps, and therefore typically acts as a redox-innocent spectator ligand. However, its significance has spurred a large amount of research into expanding upon this popular motif, and derivatives such as cyclic alkyl amino carbenes (cAAC) have been developed.^12^ These cAAC ligands feature only a single nitrogen atom and exhibit much smaller HOMO–LUMO gaps due to the absence of aromaticity (Figure 2). Consequently, metal–cAAC complexes can act as noninnocent ligands, and several carbene radical species have been reported.^13^

In combination with metal halide salts of Zn,^14^ Mn,^15^ Au,^16^ and Cu^17^ two equivalents of the cAAC ligand coordinate to the metal. Upon reduction with strong reducing agents like KC_8_ or metallic sodium/potassium, the halide is displaced and produces the highly unsaturated two-coordinate M(cAAC)_2_ complexes featuring metal centers in low oxidation states. The greater π-accepting character of cAAC ligands vs archetypical NHCs results in single-electron transfer from the metal to the carbene, yielding the carbene radical. The electron configuration of the system varies based on the type of metal used (Figure 2).

**Figure 3:**
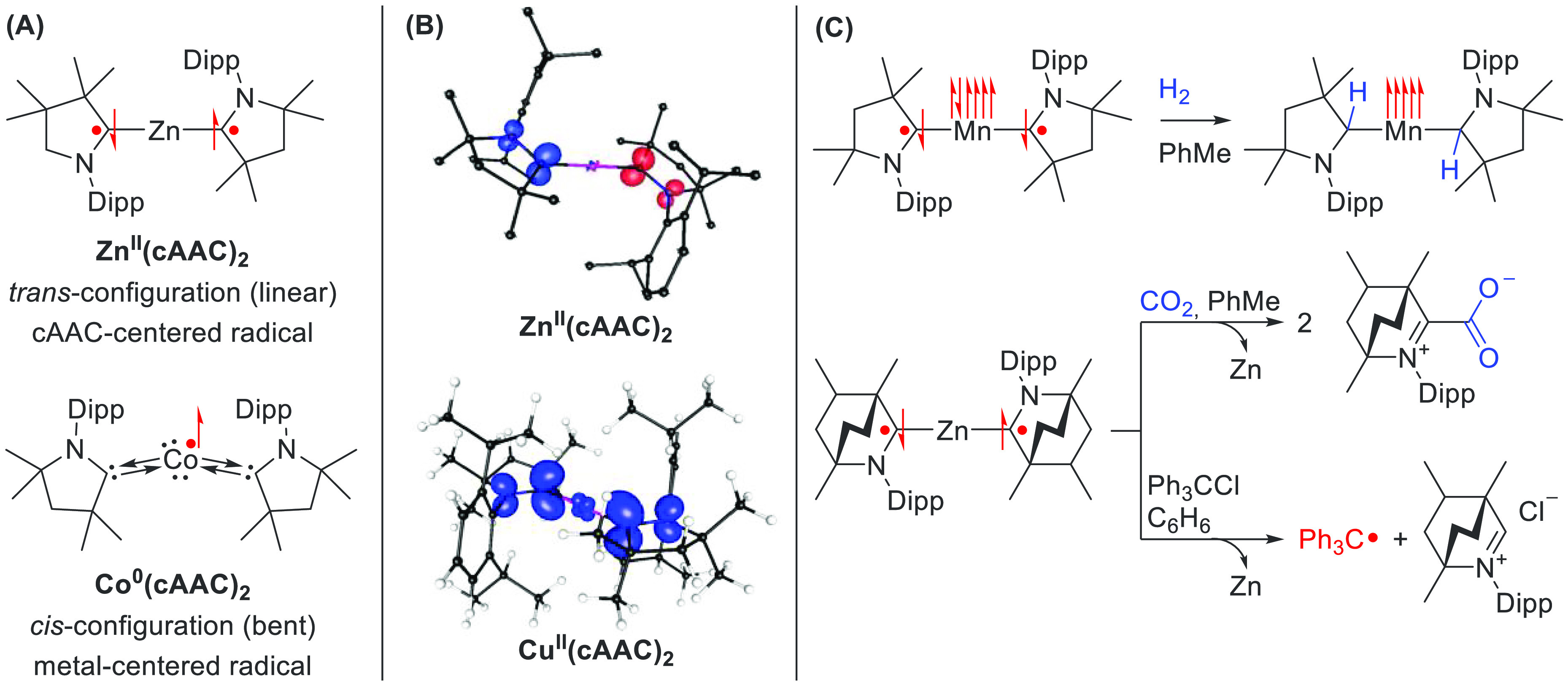
(A) *trans* (linear) vs *cis* (bent) configurations of neutral M(cAAC)_2_ complexes. The bent structures feature metal-centered radicals and neutral cAAC ligands, whereas the linear structures feature ligand-centered cAAC radicals. (B) Spin density plots of a Zn(cAAC)_2_ and a Cu(cAAC)_2_ complexes. (C) Reactivity of Mn(cAAC)_2_ complexes toward H_2_ and that of Zn(BICAAC)_2_ complexes toward CO_2_ and trityl chloride.

cAAC complexes featuring late transition metals, such as Cu^17^ and Au,^16^ are generally low-spin, and here a single unpaired electron is largely distributed over the two cAAC ligands (Figure 3A). Zn(cAAC)_2_ complexes are also low-spin, but their integer nature leads to an open-shell singlet ground state with both electrons antiferromagnetically coupled on the cAAC ligands (Figure 3B).^14^ Early transition metals exhibit intermediate- to high-spin character, leading to more complex electronic states, such as that for a Mn(cAAC)_2_ complex.^15^ Here, the major electronic ground state is a Mn(I) d^6^ configuration with one electron delocalized over the two cAAC ligands, with a significant contribution from a Mn(II) d^5^ configuration and two antiferromagnetically coupled electrons on the two cAAC ligands. Additionally, a geometric trend can be observed where electron transfer occurs from the metal to the carbene ligands when the metal–cAAC complex is linear. For bent structures, as observed in Fe,^18^ Co,^19^ Ni,^20^ Pd, and Pt^21^ analogues, this single-electron transfer does not occur. Here, the Dipp ancillary groups are in a *cis*-configuration with respect to one another and the bulky methyl groups twist the overall structure, reducing the orbital overlap between the carbene moieties. The optimal overlap for the linear structures vs the reduced overlap of the bent structures results in the observed electronic differences.

Reactivity studies were performed for the Mn^14^ and Zn^15^ complexes (Figure 3C). The Mn complex reacts with hydrogen in a fashion reminiscent of a metal–ligand cooperative system. H_2_ splitting occurred in mere seconds at ambient temperatures for the Mn complex, whereas it takes 5 h at 35 °C for the free cAAC ligand. Additional calculations reveal a low H_2_ affinity for the Mn complex (Δ*G*_298_ = −6.2 kcal·mol^–1^), suggesting its potential catalytic application. Likewise, the Zn complexes proved to be much more reactive than the free carbene. Reacting the metal complex with CO_2_ released the free carbene–CO_2_ at ambient conditions in only 1 h for the BICAAC complex and at −30 °C for the cAAC-based complex. This is peculiar, since R_2_Zn complexes normally do not react with CO_2_ at room temperature. The increased reactivity toward CO_2_ is ascribed to the radical nature of the carbene ligands. Further reactivity studies showed that the reaction of Zn(BICAAC)_2_ with trityl chloride resulted in halide displacement and single-electron reduction of the trityl moiety to produce the trityl radical Ph_3_C^•^ (Figure 3C).

## Carbodicarbenes

**Figure 4:**
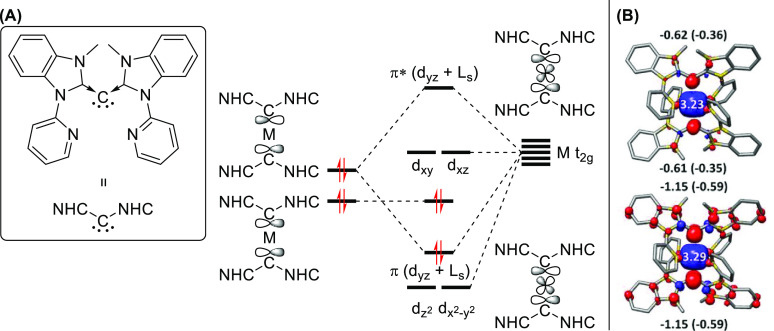
(A) General MO diagram for M(CDC)_2_ complexes (CDC = carbodicarbene). One CDC lone pair forms a σ-bond with a metal d-orbital, and another lone pair forms a nonbonding MO. At higher overall oxidation states, an electron is removed from the latter to form a Schrock-like carbene radical. (B) Spin density plots for a chromium-based CDC complex in its 4+ and 5+ states.

Carbodicarbenes (CDCs) (Figure 4) are analogous to NHCs and are essentially two carbenes “coordinated” to a carbon(0) center, resulting in a unique bent allene-like structure. In contrast to NHCs and similar stable carbenes like cAACs, CDCs have two filled carbon p-orbitals and as such are both σ- and π-donating.

The group of England investigated the redox noninnocence of such CDCs in Cr,^22a^ Fe,^22b^ and Co^22a^ complexes using Ong’s tridentate ligand^23^ with pendant pyridine moieties. Two CDC ligands coordinate to the central metal to form a homoleptic octahedral complex. Extensive spectroscopic and computational studies show that these species can be oxidized reversibly several times from an initial dicationic species to a pentacationic state.

In the dicationic [M(CDC)_2_]^2+^ state, all orbitals associated with C–M–C π-system are doubly occupied for the Co and Fe complexes. The Cr complex was found to have an electronic structure intermediate between two limiting resonance forms, where one electron is delocalized over the two carbene moieties [Cr^III^(CDC^•–^)(CDC)]^2+^ and [Cr^II^(CDC)_2_]^2+^. Upon oxidation to the tricationic [M(CDC)_2_]^3+^ state, a metal-centered oxidation occurs first for all three complexes, whereas for the second oxidation to the tetracationic [M(CDC)_2_]^4+^ state the redox event is ligand-centered. However, the electronic structure is complicated here and is best described as an open-shell singlet with strong antiferromagnetic coupling between the metal and carbene centers, producing [M^III^(CDC)(CDC^•+^)]^4+^ species. It is important to note that, for the tetracationic state, strong π-donation by the ligand cannot be ruled out entirely. Later, the experimentally unstable pentacationic state was found to feature a carbene radical in all cases, with a single unpaired electron in the nonbonding MO of the carbene ligands.

The dual σ- and π-donating properties of the CDC ligands is analogous to more conventional Schrock-type carbenes, and the relative orbital energies with respect to metal d-orbitals are also in line with Schrock-type character (throwback to the carbene MO picture). Since the HOMO of a Schrock carbene is the carbon-centered MO, removal of an electron should generate a Schrock-type carbene radical (as opposed to the addition of an electron to Fischer-type carbenes, which is more often observed). This description accurately matches the experimental and computational observations of these oxidized [M(CDC)_2_]^*n*+^ complexes. These species are classified as Schrock-type carbenes that have been oxidized by a single electron and therefore can be referred to as Schrock-type carbene radicals.

Another example of such a Schrock-type carbene radical was reported by the group of Arnold.^24^ Oxidation of a Ta(V) Schrock-type carbene by the addition of 1 equiv of AgB(C_6_F_5_)_4_ resulted in a carbon-centered radical. A spin density plot derived from DFT calculations shows a carbon-centered radical that is partially delocalized over the metallacycle.

## PC_carbene_P Pincer Complexes

**Figure 5:**
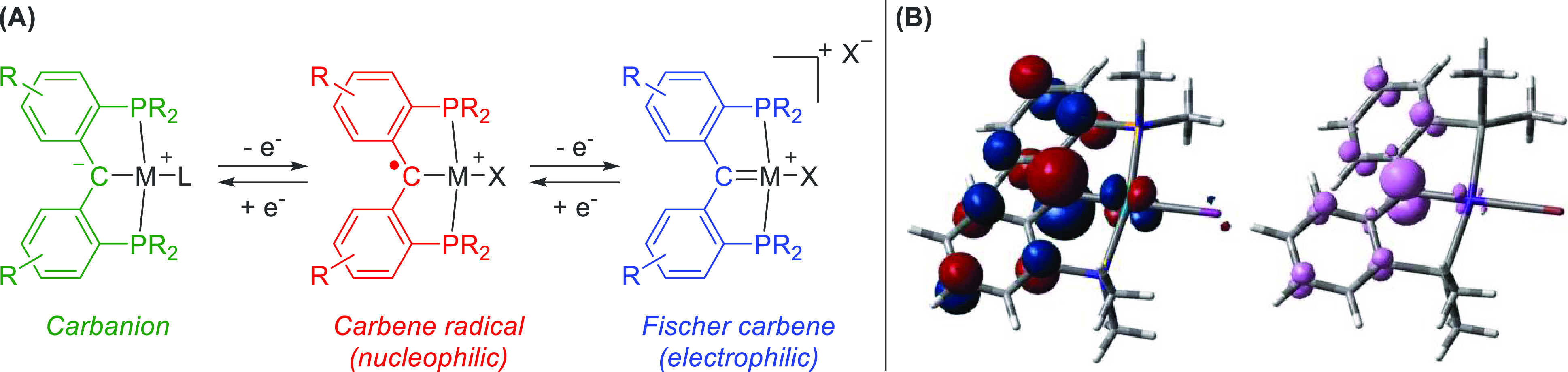
(A) Oxidation states of M(PC_carbene_P) complexes featuring the anionic state (green), the carbene radical (red), and the fully oxidized Fischer carbene (blue). (B) SOMO and a spin density plot of a Pd(PC_carbene_P) complex.

Pincer ligands are highly popular motifs that strongly bind a wide variety of transition metals while creating a relatively unsaturated metal center. This often leads to a highly reactive metal center with unique properties. With the advent of redox-active ligands, such motifs were quickly incorporated in the field of reactive pincer complexes.^25^ Consequently, carbenes as redox-active/noninnocent ligands were ideal targets. The group of Iluc has extensively studied the redox properties of such PCP pincer complexes featuring a central redox-noninnocent diarylcarbene moiety for Fe,^26^ Ni,^27^ Pt,^28^ and Pd^29^ complexes. The PCP ligand can adopt three oxidation states (Figure 5). The reduced form features a carbanion (green), which upon one-electron oxidation forms a carbene radical (red) and upon further oxidation forms a Fischer-type carbene (blue). Depending on the coordinated halide, the overall charge of the metal(PCP) complexes varies.

**Scheme 2:**
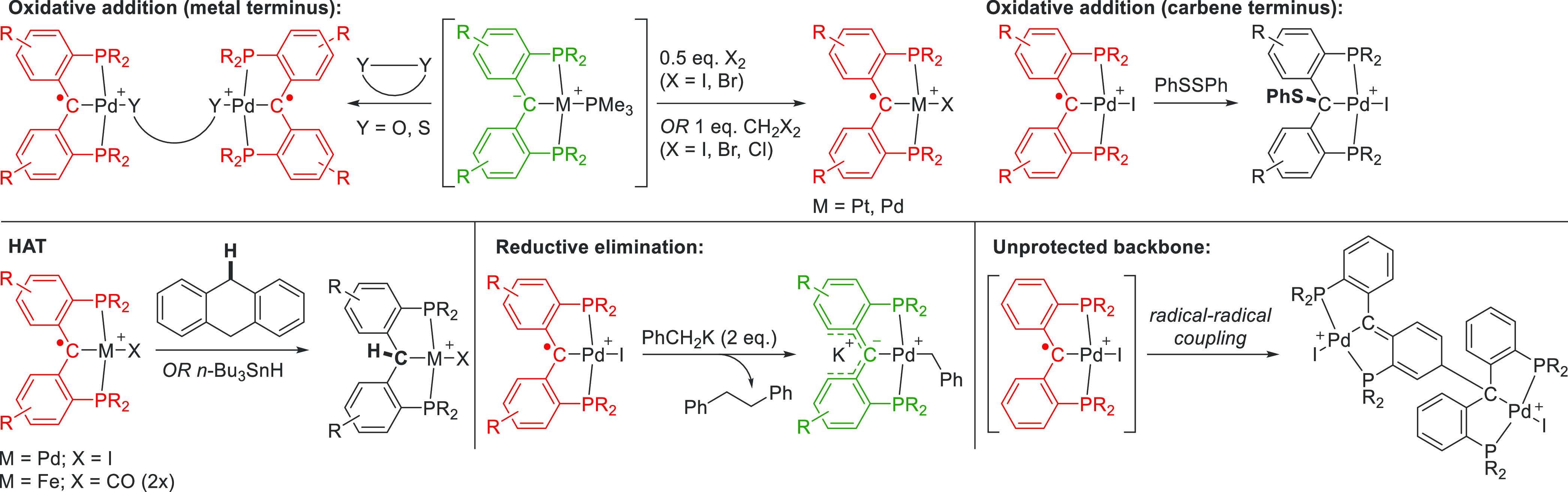
Reactivity of radical M(PC_carbene_P) Complexes Towards Oxidants, Reductants, Hydrogen-Atom Donors, and M(PC_carbene_P) Complexes Themselves

Coordination chemistry of these PCP complexes shows that the initial ancillary ligand (e.g., PR_3_, Cl, Br, or I) can be exchanged for a variety of ligands, such as amides, hydroxides, carboxylates, or alkyl species. In these instances, the ligand acts in an innocent fashion. However, in the presence of oxidants or reducing agents, the ligand reacts in a noninnocent manner. Redox reagents remove or add electrons on the central carbene ligand but do not affect the overall coordination chemistry. Coordinating oxidants such as disulfide bonds, quinones, or halogens (Br_2_, I_2_, CH_2_Cl_2_, and CH_2_Br_2_) react via oxidative addition to form anionic bridging ligands (Scheme 2), as shown for an illustrative Pd(PCP) complex. For benzyl ligands, the M–C bond can reductively eliminate to produce 0.5 equiv of the 1,2-diphenylethane dimer. Although these elementary steps are second nature for palladium and other platinum group metals, it is the redox-active carbene ligand that undergoes the oxidation state changes here.

When oxidized to its Fischer-type carbene state, the polarity of the C–M bond reverses and the carbene becomes electrophilic. Nucleophiles bind to the carbene atom instead, such as not only hydrides, ammonia, and phosphines but also nucleophilic oxidants like *N*-oxides and iminoiodinanes. The Ni(PCP) Fischer carbene is largely unreactive toward strong one-electron oxidants and small molecules (e.g., H_2_, CO, CO_2_, and N_2_O). The more electropositive iron complex can react with strong acids as well as halides (e.g., Br_2_), with the iron supplying the required electrons.

## Early Transition Metal Fischer-Type Carbenes

Group 6 Fischer carbenes (M = Cr, Mo, and W) derived from metal carbonyl complexes were the first type of carbene radical species ever described (vide supra). The organometallic properties of this branch of carbene radical chemistry has been extensively reviewed in the past.^7,30^ Expanding on this seminal work, Bezuidenhout and others elaborately explored the electrochemistry of several group 6 metal derivatives as well as other metal carbenes and carbene radicals and supported their findings with computational studies.^31^

**Scheme 3:**
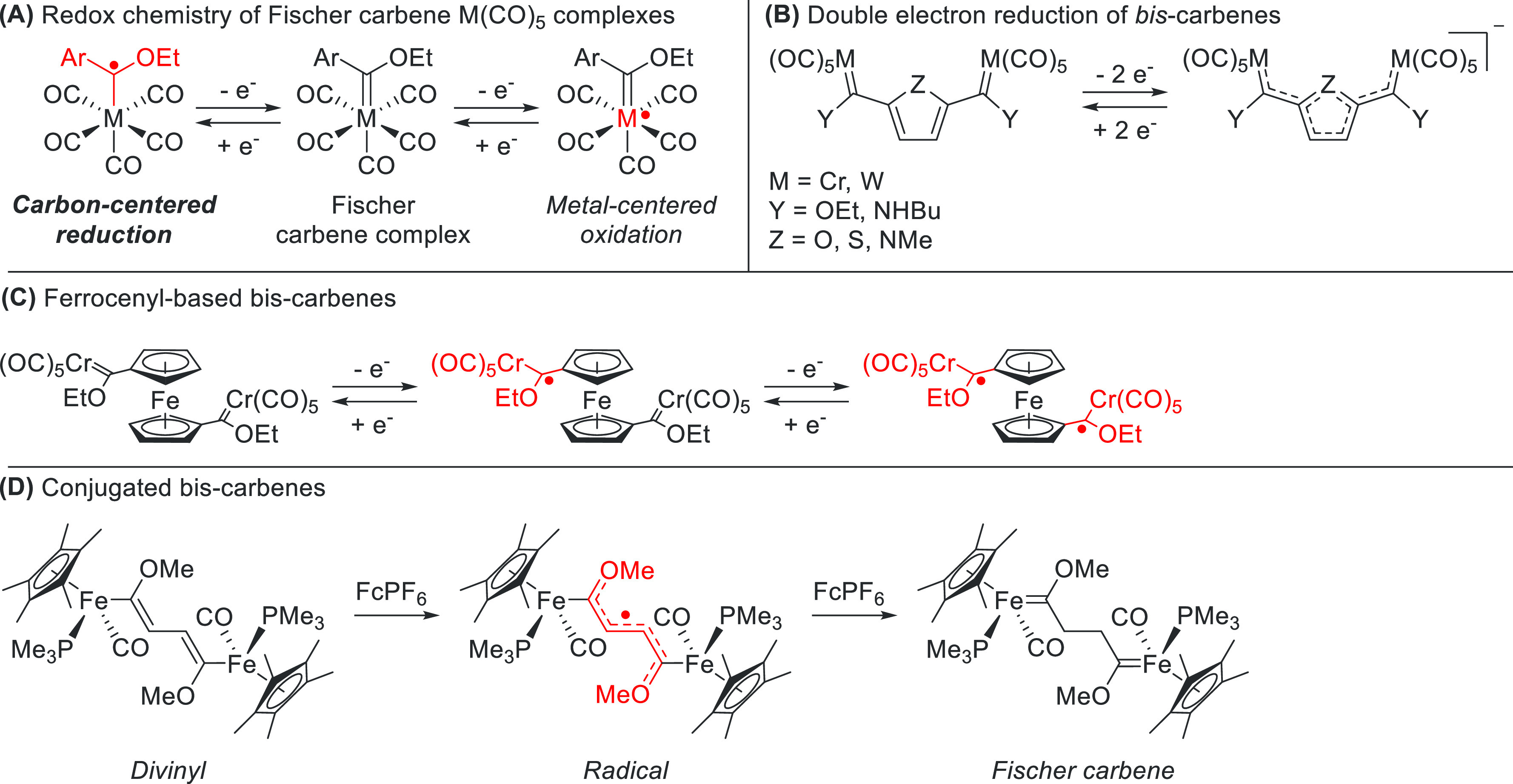
Redox Chemistry of Early Transition Metal Carbenes (A) Locus of redox events in Fischer carbene metal carbonyl complexes. (B) Electronic communication in reduced biscarbenes. (C) Electronically separated communication in ferrocenyl-based biscarbene metal carbonyl complexes. (D) Stepwise oxidation of a divinyl-bridged ferrocenyl derivative to a Fischer carbene, which proceeds via a carbene radical stage.

In general, the more electron-donating the substituents on the carbene atom, the more negative the reduction potential of the carbene ligand needed to form its radical anionic form. This applies to both aromatic and heteroatom substituents.^32^ The relation is similar for ancillary ligands on the pendant metal, where greater electron-donating capabilities or decreased π-accepting properties decrease the reduction potential of the carbene.^33^ Biscarbene derivatives were also investigated. Conjugated biscarbenes (Scheme 3) can be formed via stepwise two-electron oxidation from a divinylic species, which proceeds via a delocalized carbene-like radical.^34^ More distantly connected biscarbenes do communicate electronically, such as via a ferrocenyl group, but can also be reduced separately.^35^

## Stoichiometric Cobalt–Carbene Radical Reactivity

**Scheme 4:**
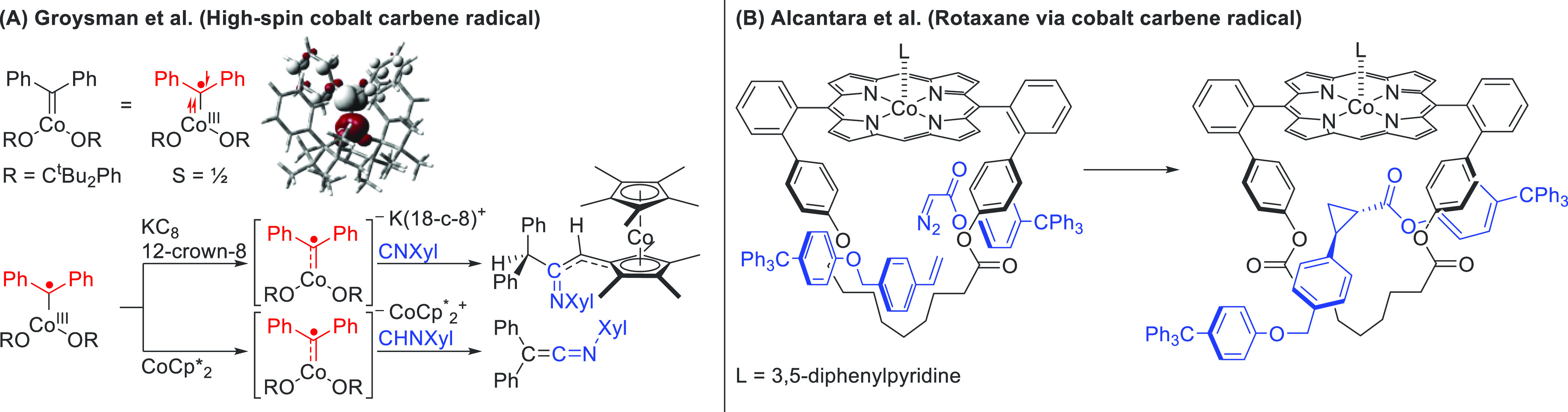
Stoichiometric Radical Reactivity of Co-Carbene Complexes (A) Formation and reactivity of a high-spin cobalt carbene complex. (B) Formation of a cyclopropane-linked rotaxane, which proceeds via a locally generated cobalt porphyrin carbene radical intermediate.

The group of Groysman developed a highly bulky alkoxide ligand that forms a biscoordinated complex with a high-spin cobalt (*S* = 3/2).^36^ In the presence of diphenyldiazomethane, a carbene is formed with an unusual broken-symmetry doublet spin state (Scheme 4A). Here, two antiferromagnetically coupled electrons are centered on cobalt and one unpaired electron is centered on carbon. This is in contrast with the low-spin (*S* = 1/2) cobalt porphyrin carbene radicals. Reduction of this high-spin carbene radical with strong reducing agents (e.g., KC_8_ and Co(Cp*)_2_) does not lead to an expected diamagnetic Co^III^–CR_2_^2–^ species but instead to a high-spin Co^II^–CR_2_^**•–**^ species featuring four unpaired electrons with three metal-centered α-spins and one carbene-centered β-spin. This overall anionic complex can react stoichiometrically with isocyanides to form ketimines and, if formed with Co(Cp*)_2_, can also react further with the metallocene reductant (Scheme 4A).

Inspired by the work of Zhang and de Bruin, Alcântara et al. used a Co^II^–porphyrinate-based macrocycle and studied the radical reactivity of Co^III^–carbenes to synthesize a porphyrin-based rotaxane. Reacting the carbene radical within the macrocyclic pocket with an alkene produced the desired rotaxane in a facile fashion in a 95% yield (Scheme 4B).^37^

## Iron: Carbene Radical or Not?

**Scheme 5:**
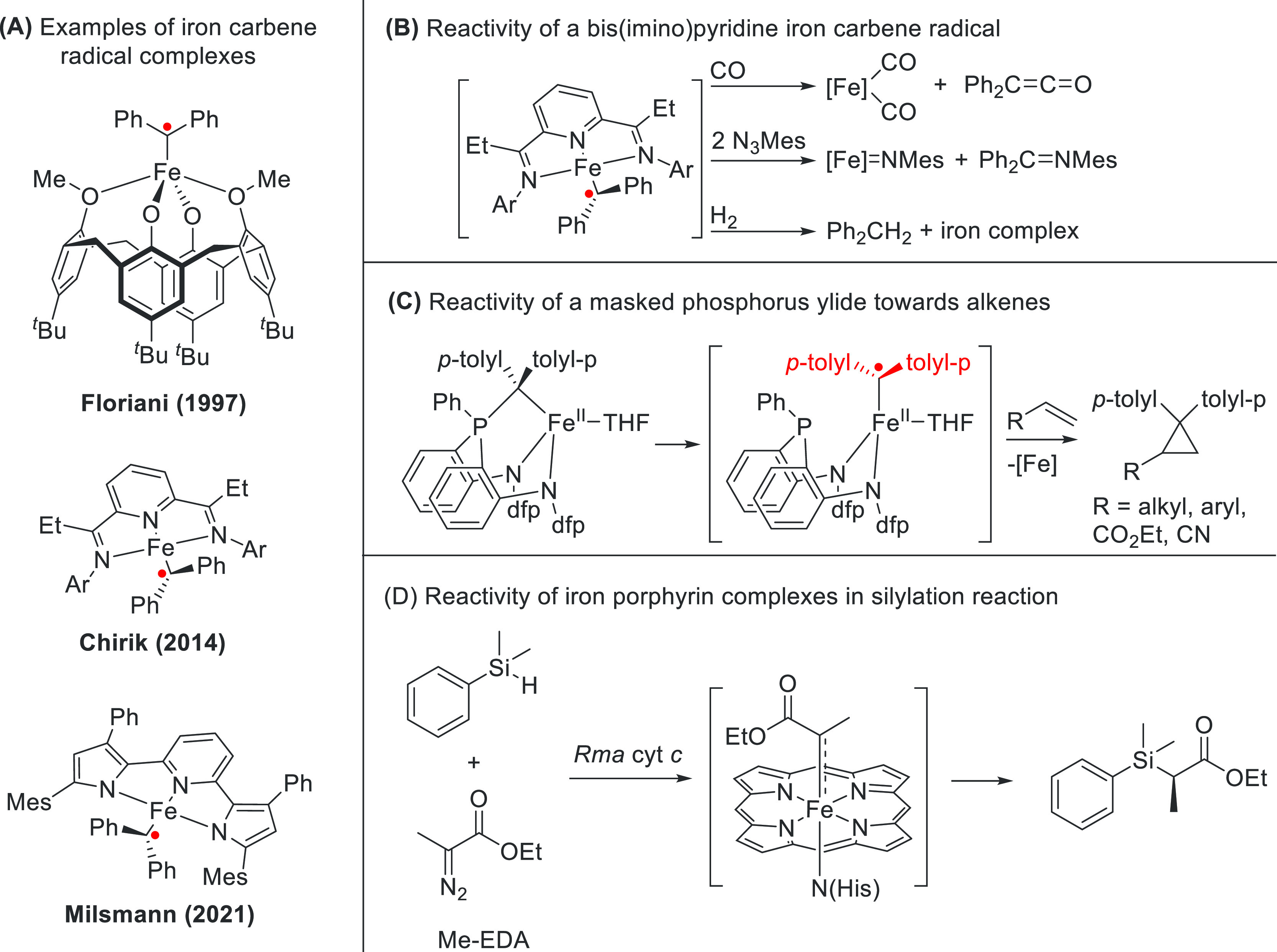
Radical Reactivity of Iron-Carbene Complexes (A) Structurally characterized reported iron carbene radical complexes. (B) Reactivity of Chirik’s iron carbene radical complex towards CO, H_2_, and azides. (C) Reactivity of a masked phosphorus ylide, proceeding via a transient high-spin iron carbene radical, that can cyclopropanate a variety of alkenes. (D) Reactivity of iron porphyrin complexes (derived from *Rhodothermus marinus* cytochrome *c*) in a carbene-transfer silylation reaction.

The diversity and success of carbene radicals and their reactivity has inspired researchers to transfer this reactivity to iron.^38^ However, this has proven to be far from trivial. The electronic nature of the iron–carbene bond and the manipulation thereof are not straightforward. Consequently, an intense discussion in the field is ongoing that centers largely around monosubstituted acceptor iron porphyrin carbenes (IPCs) derived from diazoacetates. These IPCs are best described as either closed-shell diamagnetic Fischer-type Fe(II)←{:C(X)Y} carbenes or open-shell paramagnetic Fe(III)–{C(X)Y}^•–^ (where X and Y resemble different R groups). A third, Schrock-type Fe(IV)={C(X)Y}^2–^ has also been proposed on occasion,^39^ although Wolczanski and co-workers later revised it to the Fischer-type Fe(II)←{:C(X)Y} electronic structure (Scheme 5A).^40^ Experimentally, extensive spectroscopic and stoichiometric reactivity studies of several iron carbenes have elucidated their open-shell radical electronic structure in detail (Scheme 5A).^41^

Danopoulos et al. synthesized heteroleptic M(NHC)(HMDS) complexes (M = Co, Fe) in low oxidation states. While normally NHCs, due to their large HOMO–LUMO gaps, do not exhibit significant π-accepting properties, here the high electron density on the metal leads to reduction of the NHC moiety to a carbene radical.^42^ These complexes exhibit high magnetic moments and are both high-spin complexes featuring a M^II^L^**•-**^ configuration. Other examples of radical NHC complexes were also reported by the groups of Frenking, Enders, Wang, and Apeloig.^43^

The group of Chirik investigated a bis(imino)pyridine iron carbene complex (Scheme 5B).^44^ The low oxidation state of the iron center, coupled with the redox-active nature of the bis(imino)pyridine ligand, led to an unusual broken-symmetry septet state (*S* = 1) with four α-electrons and two β-electrons. This rare example of an iron carbene radical can react stoichiometrically with CO to produce ketenes and with azides to produce both an iron nitrene complex and an imine product. In the presence of H_2_, diphenylmethane was liberated (Scheme 5B). Work by Liu et al. on a masked phosphorus ylide iron complex (Scheme 5C) revealed stoichiometric radical reactivity in the cyclopropanation of a variety of alkenes.^41d^ A combination of experimental and computational studies supports the hypothesis that the initial ylide can undergo P–C cleavage to form a metastable iron carbene radical that proceeds to react with vinylic substrates.

## Catalysis

In the field of d^6^-metalloporphyrins as catalysts for carbene transfer, iron is in the spotlight.^38b,48^ Due to its high abundance and low toxicity, it is a prime platform for developing efficient and new carbene transfer protocols. Consequently, significant effort has been made to impart a radical character onto the classical Fischer-type carbenes to unlock new mechanistic pathways. There is currently an ongoing debate whether certain iron-based carbenes as intermediates in catalysis can react and behave as carbene radicals.^49^

**Scheme 6:**
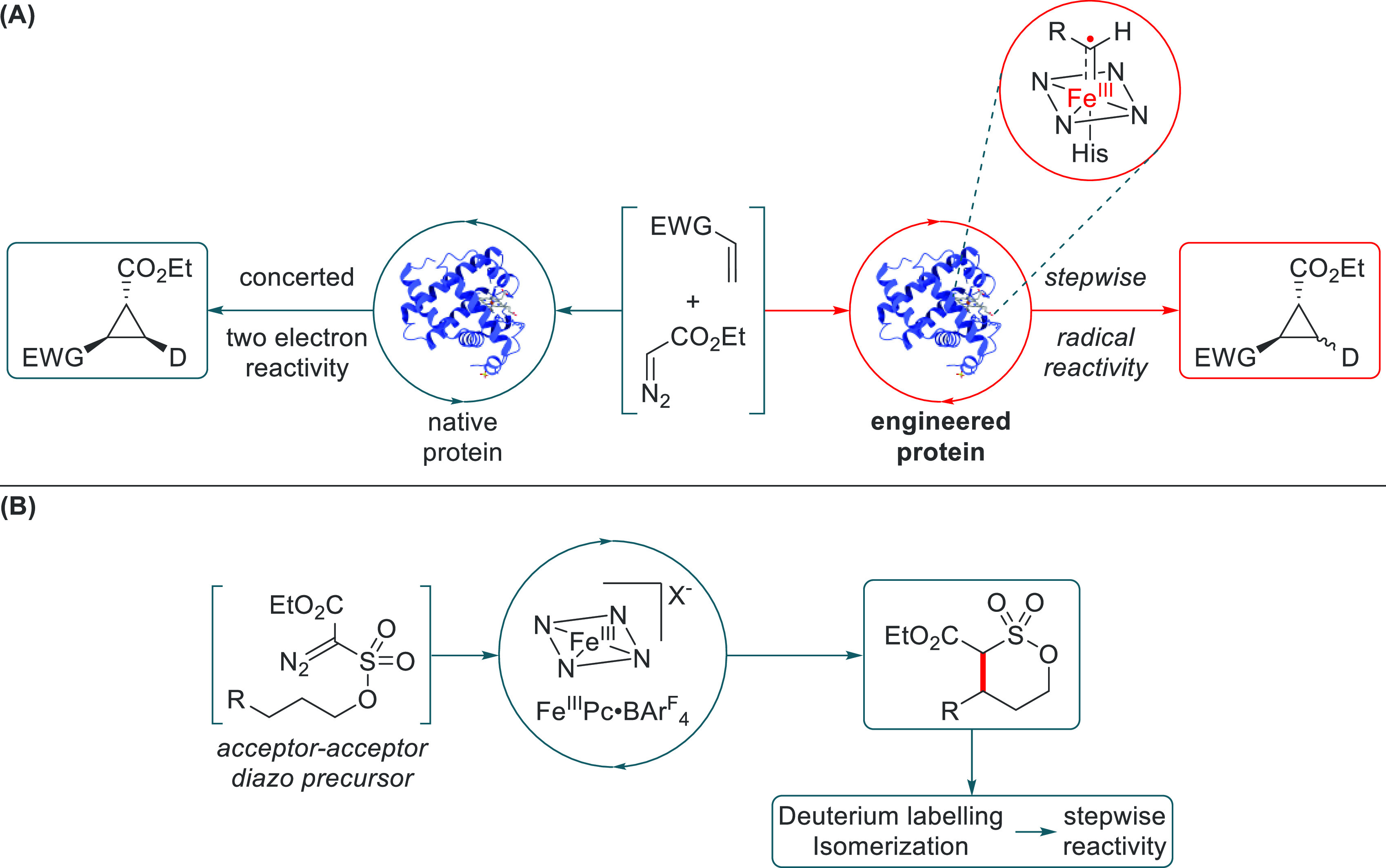
Radical-Type Transformations Catalyzed by Iron-Carbene Complexes (A) Biocatalytic approach by Fasan and coworkers that resulted in stepwise, iron-carbene radical reactivity. (B) Stepwise iron-catalyzed C–H alkylation via an acceptor–acceptor diazo precursor (Pc = phthalocyanine).

Recently, the group of Arnold reported a series of efficient carbene transfer reactions using engineered heme-type Fe enzymes (Scheme 5D).^46^ Expanding on computational work by Sharon et al., the carbene intermediates involved in these reactions were proposed to be carbene radicals based on DFT studies.^49i^ The presented experimental data, however, all point to electrophilic Fischer-type carbene reactivity.^46c,49c^ The proposed radical character of the iron porphyrin carbenes (IPCs) is therefore purely based on DFT studies, which were recently shown to provide an incomplete representation of the electronic structure of IPCs. On this same premise, other groups have also concluded that IPCs would have an open-shell singlet ground state.^47^ However, as shown by the group of Stroscio, the electronic structure of IPCs is best described by multireference post-HF methods, which reveal a predominantly closed-shell ground state with a small, albeit significant, contribution of the open-shell singlet state.^49j^ As such, it is clear that hybrid DFT methods overestimate the open-shell singlet character of IPCs, and these species thus seem to be best described as Fischer-type carbenes, in good agreement with their most commonly observed reactivity. An interesting exception is a recent report by Fasan and co-workers, showing that specific enzyme modifications can apparently enhance the (open-shell singlet) radical character of IPCs, giving rise to discrete (experimental) carbene radical behavior. This was observed for an engineered myoglobin protein Mb(H64V, V68A) containing a native heme factor with a noncanonical *N*-methyl histidine axial ligand (Scheme 6A).^50^ This alternative protein was revealed to react in a stepwise radical fashion in contrast to unaltered conventional myoglobin carbene transferases. Using *cis*-β-deuteriostyrene, a 1.2:1 *cis*/*trans* ratio was observed for the altered protein whereas the native proteins gave no isomerization whatsoever. Additionally, kinetic Hammett studies revealed that the best fit for the altered protein was a combination of polar- and spin-delocalization Hammett parameters and could be described as a nucleophilic radical. As expected, data for the native transferases best fit the positive σ-parameter in the absence of a radical parameter, in line with a nonradical electrophilic carbene. Inhibition experiments with the radical spin trap 5,5-dimethyl-1-pyrroline *N*-oxide (DMPO) yielded results further supporting the previous findings, where the altered transferase showed a significant degree of inhibition while the native proteins did not.

White and co-workers investigated the intramolecular C–H insertion of acceptor–acceptor diazo compounds into active C–H bonds using an iron(III) phthalocyanine (Scheme 6B).^51^ With isotopic labeling of the active C–H position, they showed a large KIE of 5.0, which is suggestive of a stepwise radical mechanism. Substitution of the pendant phenyl group with a vinyl moiety revealed that significant isomerization occurs for the [Fe(III)Pc] catalyst, whereas isomerization barely occurs for [Rh_2_(OAc)_4_] (a catalyst established to operate via a concerted mechanism). However, the presence of a catalytically operative metal carbene radical intermediate was not directly proven.

**Scheme 7:**
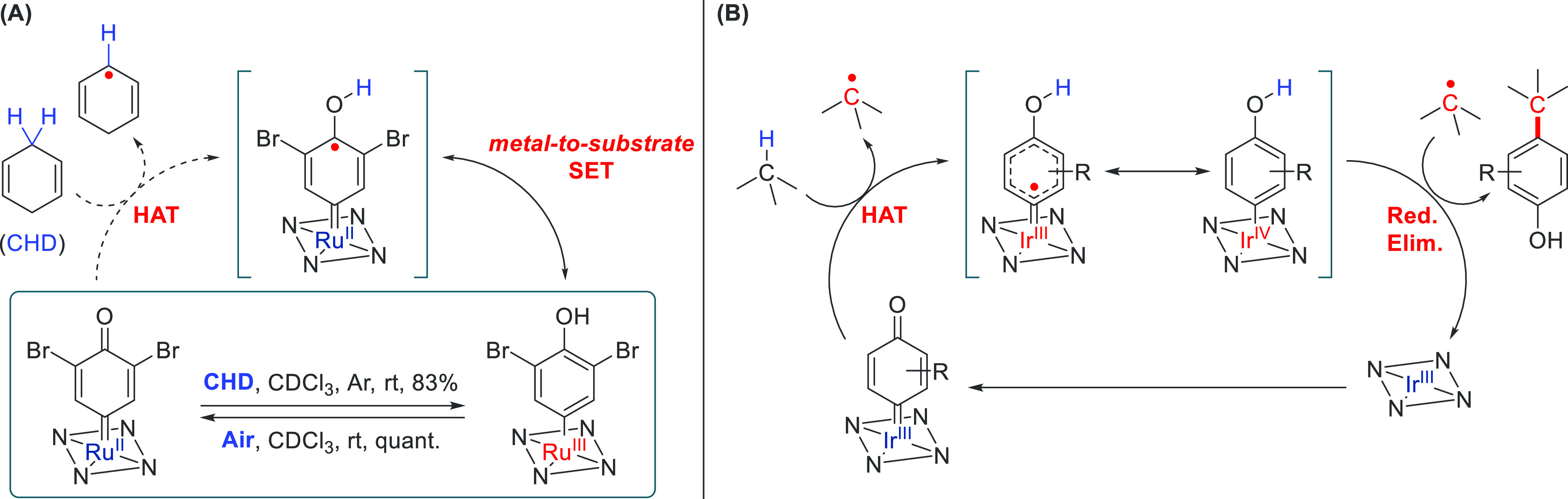
Radical Reactivity of Ru- and Ir-Carbene Complexes (A) Stoichiometric Hydrogen Atom Transfer (HAT) with Ru–quinoid carbenes (QCs), followed by metal-to-substrate electron transfer and subsequent oxidative aerobic regeneration. (B) Catalytic stepwise C–H insertion via hydrogen atom transfer, followed by reductive elimination to liberate C–H-inserted phenols.

Conventionally, d^6^-metalloporphyrins proceed via two-electron concerted pathways when operating as carbene-transfer catalysts. Investigating the chemistry and catalytic application of diazo quinones, the group of Che discovered that the incipient Ru and Ir carbenes proceed via unique radical stepwise pathways in C–H insertion reactions (Scheme 7).^52^

Initial stoichiometric Hammett studies on the Ru–quinoid carbenes (QCs) and their reaction with nitrosoarenes showed a preference for more electron-donating groups on the *meta*-substituent of the QCs.^52a^ This is in contrast with conventional Fischer-type Ru carbenes, where rate acceleration is observed for electron-withdrawing substituents. Applying these Ru-QCs in stoichiometric hydrogen atom transfer (HAT) reactions, the authors found that cyclic 1,4-dienes could be converted to their aromatic dehydrogenated counterparts under mild conditions. Reaction of the QC with a HAT-donor like 1,4-cyclohexadiene produced a paramagnetic Ru(III) species, Ru–QC–H^•^, as shown by NMR and ESI-MS studies (Scheme 7A, top). Upon exposure to air, this paramagnetic species reformed the initial Ru-QC species. Based on these findings, Che and co-workers applied the Ru system catalytically. Exploiting the dual reactivity of the Ru–QCs, a variety of *p*-nitrosoarenes and *m*-disubstituted QCs could be converted to nitrones. Under aerobic conditions, exploiting the regeneration of the paramagnetic Ru–QC–H^•^, they were able to catalytically dehydrogenate 1,4-cyclohexadiene to benzene with a maximum TON of 48.

Building on the lessons learned for the Ru-based system, the Che group surmised that the use of a more electron-poor metal should prevent the reductive quenching of the incipient QC–-H^•^ radical and allow for radical rebound to form the C–H insert product.

Indeed, when switching from the more electron rich Ru(II) porphyrin to a more electron poor Ir(III)–Me porphyrin, diazo quinones produced C–H inserted products in the presence of 1,4-cyclohexadiene and catalytic Ir porphyrin (2 mol %).^52b^ The reaction was found to be highly sensitive to steric influences.

The most efficient catalyst was void of any *meso*-substituents on the porphyrin, and the flat octaethylporphyrin gave the highest yields (62%). Substitution on both the quinoid carbenes and the HAT donor next to the insertion point proved to affect both the yield and regioselectivity. α-Substitution of 1,4-cyclohexadiene gave a 11:1 ratio, favoring the nonsubstituted position for C–H insertion. The proposed radical mechanism was supported by trapping experiments using (2,2,6,6-tetramethylpiperidin-1-yl)oxyl (TEMPO), kinetic isotope effects using THF/THF-*d*_8_, and EPR measurements of a trapped Ir-QC-H^•^ radical (Scheme 7B, top).

**Scheme 8:**
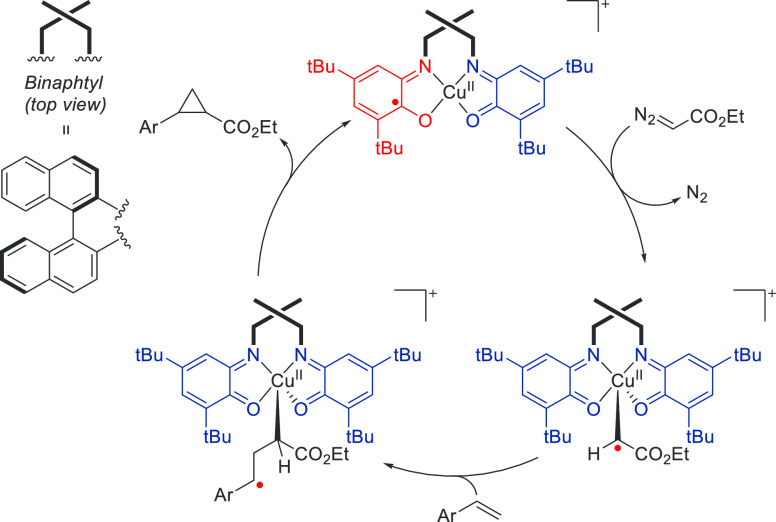
Ligand-to-Substrate SET from a Redox-Active *o*-Aminophenolate-Based Copper Complex to a Reactive Carbene Moiety and Its Radical Reactivity with Styrenes

Although the formation of a catalytically competent carbene radical is often accomplished by SET from the metal to the carbene, the group of Desage-El Murr employed the use of a redox-active ligand in combination with a copper metal center (Scheme 8).^53^ Copper has already been used to great effect in contemporary carbene transfer catalysis but generally has been observed to operate via two-electron concerted mechanisms.^54^ Through use of two redox-active *o*-aminophenolate moieties bridged by a chiral binaphthyl group, the Desage-El Murr group has shown that their Cu-catalyzed cyclopropanation of styrenes likely operates via a stepwise radical mechanism.

While the analogous nitrene transfer experiments also performed yielded more a conclusive radical character by virtue of EPR measurements, additional DFT calculations indicated that both the Cu carbene and Cu nitrene exhibit a similar radical character.

Electron transfer occurs from the redox-active ligand to the carbene (or nitrene) moiety, which initially resides in a semi-iminoquinone-benzoiminoquinone state and after SET is transformed into a bis(benzoiminoquinone) state. After the reaction with styrene, a γ-radical is produced, which in turn releases the cyclopropane after a radical rebound reaction. To the best of our knowledge, this was the first example of a catalytic ligand-to-carbene SET reaction applied in catalysis.

## Cobalt–Carbene Radicals

**Figure 6:**
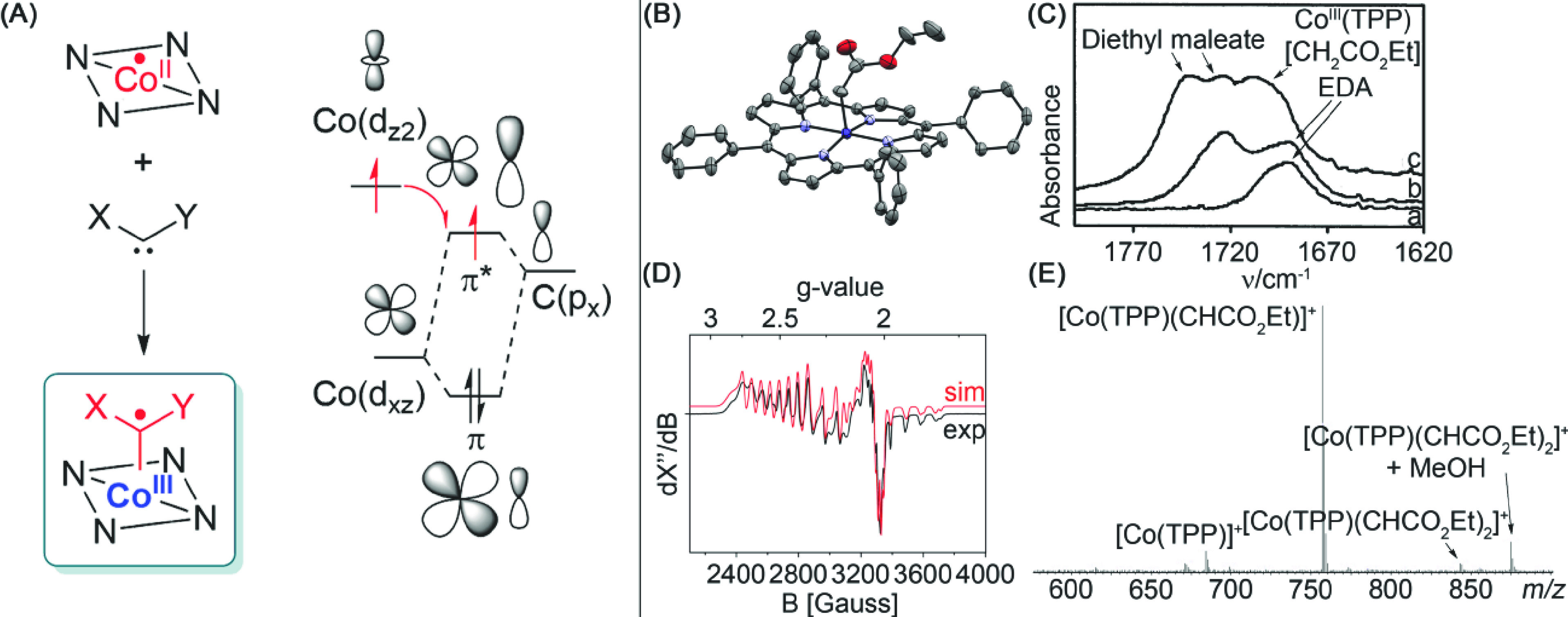
(A) Single-electron transfer from a high-energy Co-d_*z*^2^_ orbital to the LUMO of the Co–C carbene π-bond, generating the carbene radical. (B) ORTEP drawing of [Co^III^(TPP)(CH_2_CO_2_Et)]; thermal ellipsoids are drawn at 30% probability. (C) FT-IR detection of [Co^III^(TPP)(CH_2_CO_2_Et)]. (D) X-band EPR detection of the [Co^III^(TPP)(C·HCO_2_Et)] radical, along with the bridging adduct and the free [Co] catalyst. (E) ESI-MS (positive mode) detection of [Co^III^(TPP)(CHCO_2_Et)]^+^.

By and large the most abundant catalytic application of carbene radicals involves cobalt(II)-based catalysts, comprising ∼90% of the current literature. The electropositivity of cobalt is perfectly balanced between middle and late transition metals, and in its d^7^-configuration cobalt has a high-energy electron that can easily be transferred to the more electronegative carbene acceptor to produce carbene radicals (Figure 6).

Of these cobalt-systems, the most prevalent and successful platforms are tetraarylporphyrin-based catalysts. In the early 2000s, mechanistic insight on Co(II)-based carbene transfer began to grow, focusing largely on cyclopropanation as the reaction of choice. The group of Zhang showed that use of Co(II) porphyrins enabled them to effectively cyclopropanate electron poor alkenes, which are difficult to convert using conventional Fischer-type carbenes.^55^ Moreover, there was no need for the use of slow addition to prevent dimerization products. Penoni et al. studied the reaction of ethyl diazoacetate and styrene using a combination of kinetic studies and spectroscopic measurements (IR, NMR, and XRD) (Figure 6).^10a^ Under their reaction conditions, no dimerization products were observed, which is in line with the findings of Zhang and co-workers. Based on kinetic studies and IR measurements, these cobalt carbenes bridge with the porphyrin nitrogen atoms. However, this behavior is also observed for conventional Fischer-type carbenes. In absence of styrene, the carbene reacted to form a Co(III)–alkyl adduct that was structurally confirmed by XRD (Figure 6B), which can only be explained by reaction of the carbene with a hydrogen atom. FT-IR studies and complementary calculations by the group of Yamada for metal carbenes revealed that the carbonyl IR shift of the pendant ester groups was highly dependent on the oxidation state of the metal carbene.^56^ Co–salen- and Co–porphyrin-based carbene complexes exhibited markedly lower absorption wavenumbers for the C=O stretching band than conventional Fischer-type carbene complexes. Complementary calculations postulated that this is due to reduction of the carbene and that spin delocalization into π*-orbitals of the pendant ester moiety shifts the C=O band to lower wavenumbers.

More conclusive evidence was obtained by Dzik et al., who used a combination of X-band EPR, ESI-MS, and DFT-calculations to further elucidate the radical character of the carbene and the stepwise reactivity with styrene (Figure 6).^57^ The EPR measurements of the reaction of EDA with a chiral H-bonding cobalt porphyrin catalyst gave a mixture of a hydrated catalyst, including a bridging carbene and an organic radical species, which were observed by Penoni et al. (vide supra).^10a^ This last species was determined to be a terminal cobalt(III)–carbene radical. Additional ESI-MS measurements confirm the detection of the monocarbenoid species [Co(TPP)(CHCO_2_Et)]^+^ as well as the analogous species for [Co(3,5-di-*t*-Bu-ChenPhyrin)(CHCO_2_Et)]^+^.

DFT calculations on a model [Co^II^(Por)] (a simplified porphyrin without phenyl substituents) catalyst reveal that the reaction proceeds via a stepwise radical pathway as opposed to the conventional concerted pathway observed for Fischer-type carbenes. Access to biscarbenoid species was ruled out on the basis of inaccessible barriers as well as the lack of any dominant presence in ESI-MS measurements. The “bridging-carbene” was shown to be an off-cycle resting state for the Co(II)–porphyrin system.

**Scheme 9:**
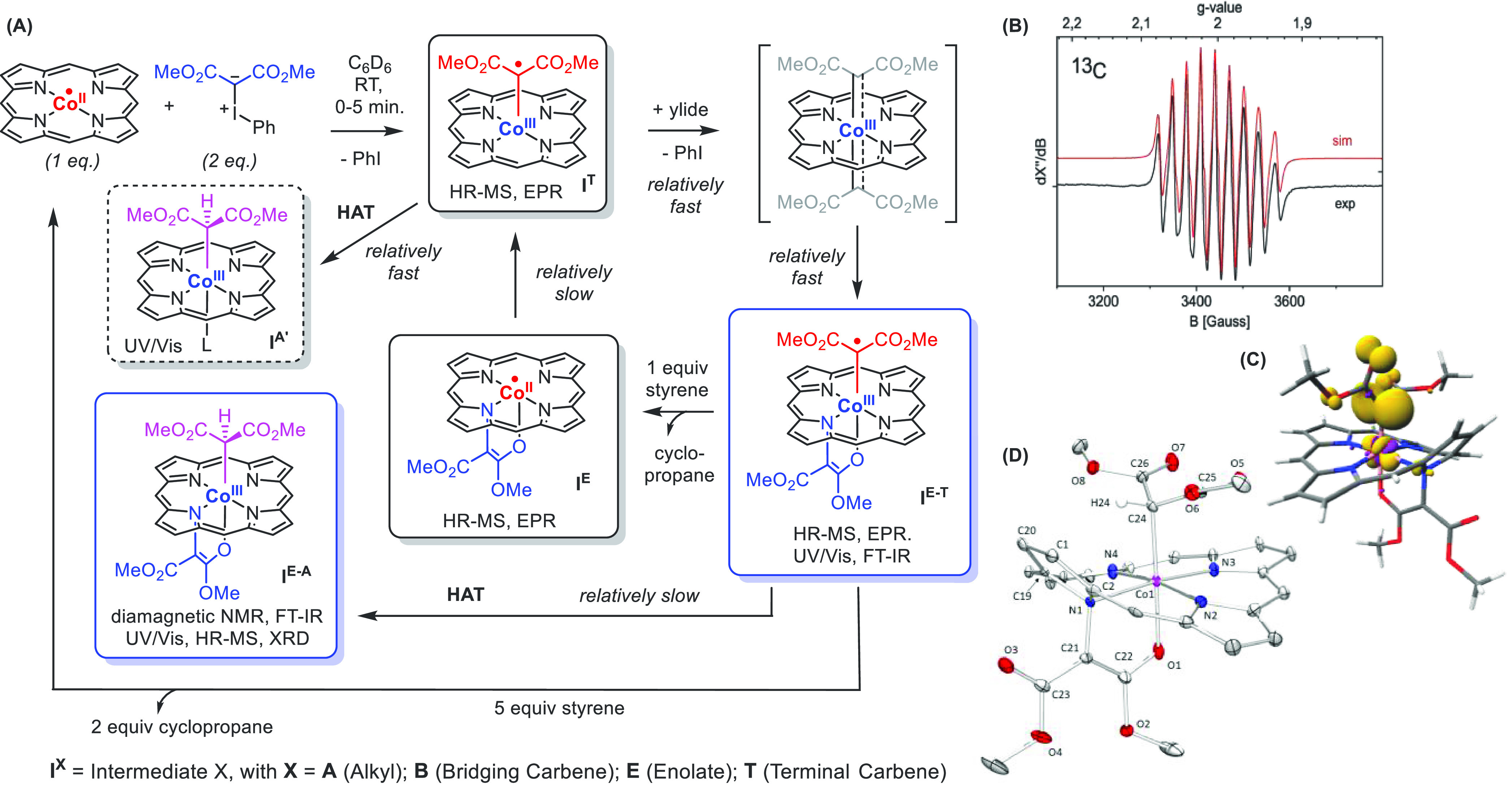
[Co(TPP)]-Catalyzed Carbene Transfer Catalysis Using Bisester-Substituted Iodonium Ylides as Carbene Precursors (A) Mechanistic and spectroscopic studies of Co(TPP)-catalyzed carbene transfer catalysis using bisester-substituted iodonium ylide as carbene precursors, showing the formation of relatively stable *N*-enolate carbene radical intermediates **I**^**E-T**^ capable of transferring both “carbene” moieties to styrene. (B) Room temperature EPR spectrum of **I**^**E-T**^ recorded in an isotropic solution of the bis-^13^C-carbene-labeled isotopic isomer of **I**^**E-T**^, showing hyperfine coupling with cobalt (*I* = 7/2) and a single ^13^C nucleus (*I* = 1/2). (C) DFT-cacluated spin density distribution in **I**^**E-T**^. (D) Structure of alkyl species **I**^**E-A**^ as derived from X-ray diffraction studies.

Additional mechanistic insight and proof for the carbene radical intermediate was obtained as well with direct detection via EPR spectroscopy or indirectly via trapping studies.^58^ Recently, Epping et al. reported elaborate and the perhaps most convincing evidence for the involvement of carbene radical species in cobalt(II)-catalyzed carbene transfer reactions using iodonium ylides as disubstituted acceptor–acceptor carbene precursors.^59^ Since iodonium ylides are more powerful carbene-delivering precursors than diazo compounds, both “monocarbenoid” (cobalt(III) monocarbene radical **I**^**T**^) and “biscarbenoid” (cobalt(III) *N*-enolate carbene radical **I**^**E-T**^) species could be detected under the catalytic reaction conditions (Scheme 9A). Transfer of two carbene units to the catalyst leads to formation of N-enolate carbene radical **I**^**E-T**^ (Scheme 9B). EPR studies of this species using the ^13^C-labeled iodonium ylide clearly revealed that the unpaired electron of these species is located at the carbene carbon atom, leading to a hyperfine interaction (HFI) with cobalt (*I* = 7/2) and a *single* carbon atom (*I* = 1/2). No HFI was detected with the ^13^C-labeled N-enolate moiety. Further evidence was obtained by spectroscopic (NMR) characterization and single crystal X-ray diffraction studies (Scheme 9D) of the alkyl species **I**^**E-A**^, which is easily obtained from carbene radical **I**^**E-T**^ by HAT from toluene.^59^

Due to their unique electronic structure, the *N*-enolates are in equilibrium with carbene radical intermediates and hence their formation is reversible. The *N*-enolate even has a protective function, slowing catalyst deactivation via hydrogen atom transfer (HAT) from the solvent. Catalytic transfer of both “carbenoid” moieties of **I**^**E-T**^ to C=C bonds is possible, which proceeds via a complex catalytic mechanism involving two interconnected cycles (Scheme 9A). The reactive paramagnetic cobalt(III) monocarbene radical (**I**^**T**^) and cobalt(III) *N*-enolate carbene radical (**I**^**E-T**^) intermediates involved in these reactions are characterized using a combination of several spectroscopic/spectrometric techniques, experimental design, computational modeling, and trapping experiments (Scheme 9A).^59^

Remarkably, the “monocarbenoid” species **I**^**T**^ do not form stable *N*-enolate isomers, while the “biscarbenoid” species are most stable as cobalt(III) *N*-enolate carbene radical species **I**^**E-T**^; as a result, *N*-enolate formation is reversible. Since the “biscarbenoid” cobalt(III) *N*-enolate carbene radical complex **I**^**E-T**^ has a reduced tendency to undergo catalyst deactivation via HAT when compared to the “monocarbenoid” species **I**^**T**^, the observed “over-carbenation” by iodonium ylides is beneficial for catalysis. The interplay of opposing stabilities between terminal and *N*-bridging carbene radical moieties dictated by the Co^II^/Co^III^-redox cycle thus plays an important role and explains the unique active participation of the otherwise catalytically inactive *N*-enolate species.

**Scheme 10:**
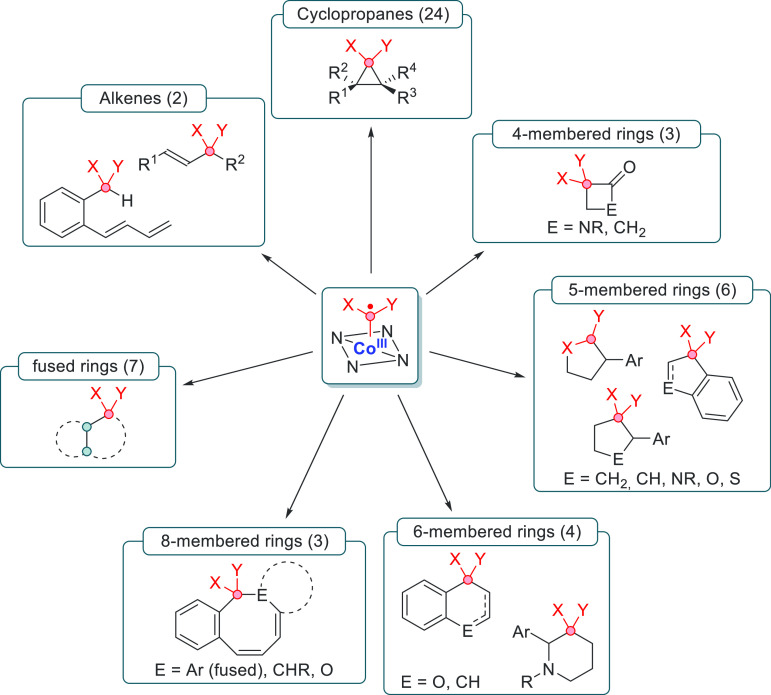
Current State-of-the-Art of the Applications of the Cobalt(III)–Carbene Radical Species Sorted Per Main Category The number of papers per category is shown in parentheses.

Application of cobalt(III)–carbene radicals has seen an enormous increase in the past decade. The effectiveness of this method has led to the successful synthesis of numerous hetero- and carbocyclic structures (Scheme 10).

Based on the accumulated research thus far, three main distinct pathways can be identified via which these carbene radicals can react: carbene addition to π-bonds, net insertion of carbenes into C–H bonds via HAT and radical rebound, and reaction mechanisms involving HAT and the formation of *ortho*-quinonedimethane (*o*-QDM) intermediates showing follow-up reactivity. These reactivity patterns are discussed in the following sections.

### Addition to π-Bonds

Taking cyclopropanation as a model reaction, the cobalt(III)–carbene radical is exceptionally adept at reacting with (delocalized) π-bonds such as aryl alkenes (Scheme 13).^60^ Addition occurs stepwise, generating a γ-radical intermediate that is stabilized by a pendant delocalizing group such as a phenyl moiety. The thus generated γ-radical subsequently attacks the σ*-orbital of the Co–C bond in a so-called “radical rebound” step. This produces the cyclopropane product and releases the Co(II) catalyst. Several related reactions leading to different products have been reported, which also proceed via carbene radical addition to π-bonds, usually followed by a radical rebound step. As such, several catalytic reactions have been developed (some of which take place in supramolecular cages providing a bioinspired protective environment around the catalyst) for the synthesis of a variety of cyclopropanes,^61^ furans,^62^ β-lactams,^63^ and numerous fused ring systems.^64^

**Scheme 11:**
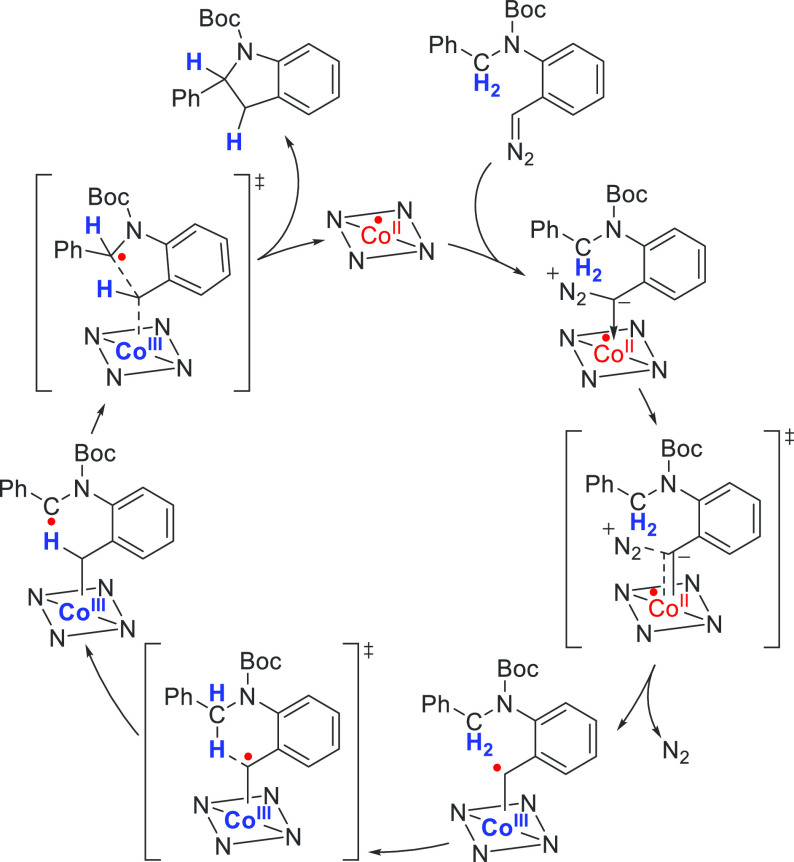
Mechanism of the Cobalt-Catalyzed C–H Insertion of an *o*-Diazoaniline to Form a Cyclized Indoline Product The square planar porphyrin ligand employed in this reaction is depicted schematically as an NNNN square.

### C–H Insertion via HAT

Fischer-type carbenes can insert into C–H bonds in a concerted manner via the interaction of the filled C–H σ-orbital with the M=C carbene LUMO.^65^ Cobalt(III)–carbene radicals react differently with C–H bonds: the interaction of the π* Co–C SOMO with the empty C–H σ*-orbital leads to HAT from C–H bond to generate a carbon-centered radical. The latter can then react via a radical rebound step by attacking the Co–C σ*-orbital, leading to the homolytic cleavage of the Co–C bond to produce the C–H insertion product and regenerating the catalyst. Since in these reactions a C–H single bond is cleaved, *inter*molecular C–H insertion reactions proceeding via these radical-type reactions result in the formation of free organic carbon-centered radicals unbound to the catalyst. Escape of free carbon radicals from the solvent cage can easily take place in undesired radical pathways, and as a result such intermolecular reactions are often not effective. However, several highly effective reactions have been developed that proceed via *intra*molecular radical-type C–H insertion reactions for which the distal carbon-centered radical cannot escape. In an intramolecular reaction, radical rebound is usually much faster than any undesired radical reaction, making the process highly efficient. Multiple examples exist, and this method has been successfully applied in the synthesis of cyclobutanones,^66^ indolines,^67^ pyrrolidines,^68^ piperidines,^69^ sulfones,^70^ and dihydronaphthalenes^71^ (Schemes 10 and 11).

**Scheme 12:**
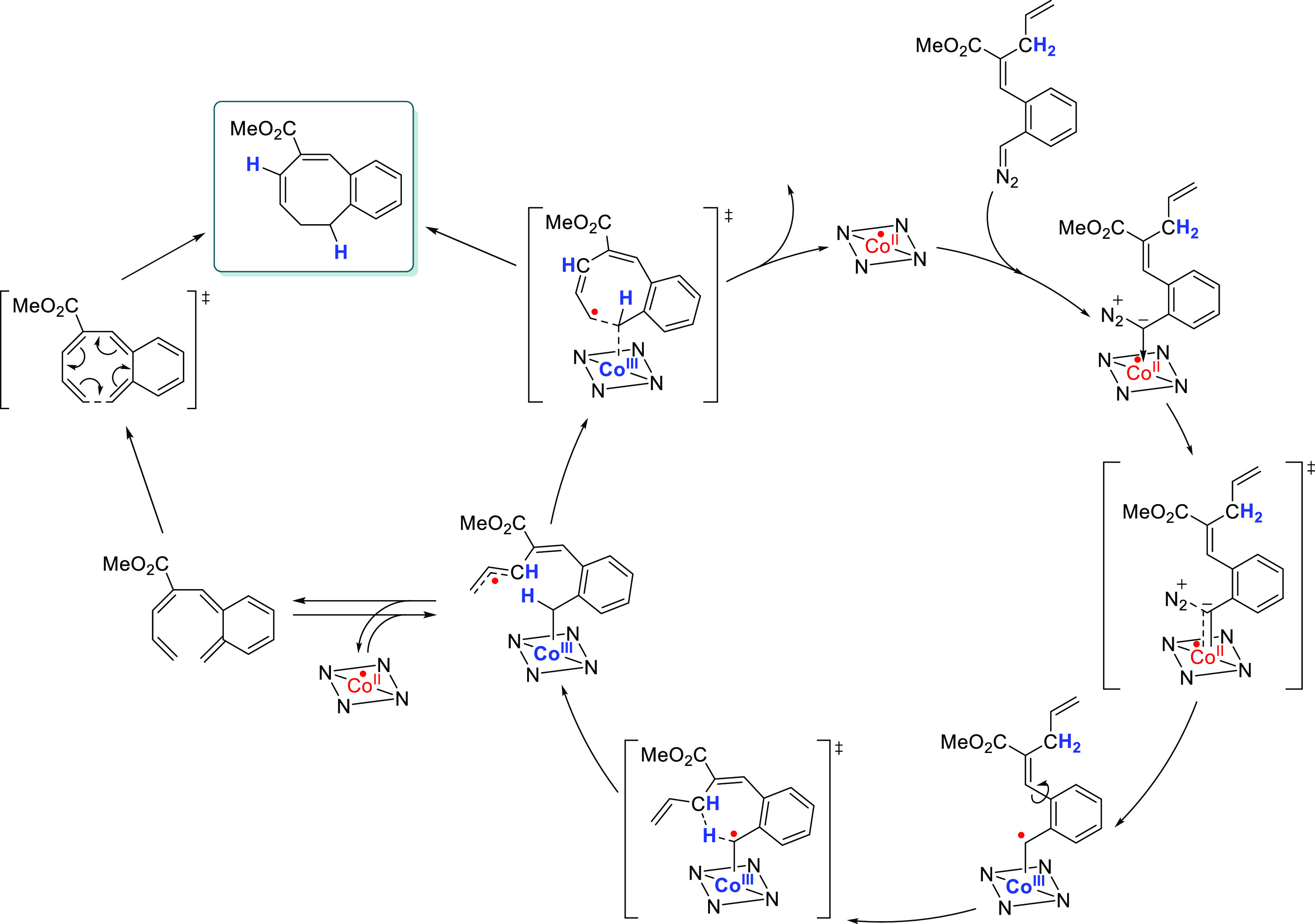
Catalytic Cycle for the Formation of Benzocyclooctenes by Cobalt(II)–Tetraphenylporphyrin Tetraphenylporphyrin is depicted here with the NNNN square. The pathway can diverge after the formation of the distal radical to form the eight-membered ring product via an electrocyclization reaction or via a radical rebound step.

### *ortho*-Quinone Dimethane Pathways

Carbene radicals with pendant *o*-phenolate or *o*-arylvinyl moieties were found to react in a peculiar manner. For these substrates, intramolecular carbene radical addition to a π-bond, or intramolecular HAT from a C–H bond in the substrate to the carbene radical, produces a distant carbon-centered radical that is in conjugation with the weak Co–C bond of the thus generated intermediate. Two pathways are now possible. The first one is via the aforementioned radical rebound mechanism, which (depending on the substrate) can be preceded by an additional intramolecular HAT reaction that results in a more favorable position for the radical rebound step (Scheme 12). The second pathway is the cleavage of the Co–C bond (in direct conjugation with the carbon radical, resulting in additional weaking of this bond) to release an *o*-quinonedimethane or *o*-quinone methide intermediate. This organic intermediate can then proceed via an electrocyclization step to form the product. This was shown to be the predominant pathway to form synthetically challenging dibenzocyclooctenes, and the use of such *o*-quinone methide/dimethanes has been used to unlock 2*H*-chromenes,^72^ 1*H*-indenes,^73^ mono- and dibenzocyclooctenes,^58c,74^ and benzoxocins.^75^

**Scheme 13:**
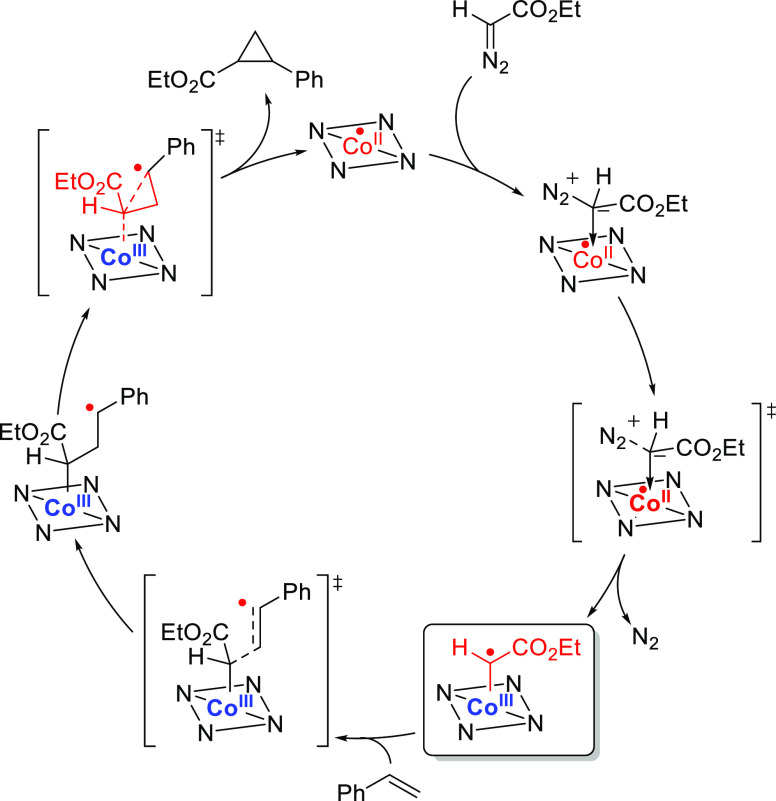
Mechanism of the Cobalt-Catalyzed Cyclopropanation of Styrene with Ethyl Diazoacetate The square planar N-donor ligands employed in this reaction are depicted schematically with an NNNN square.

**Scheme 14:**
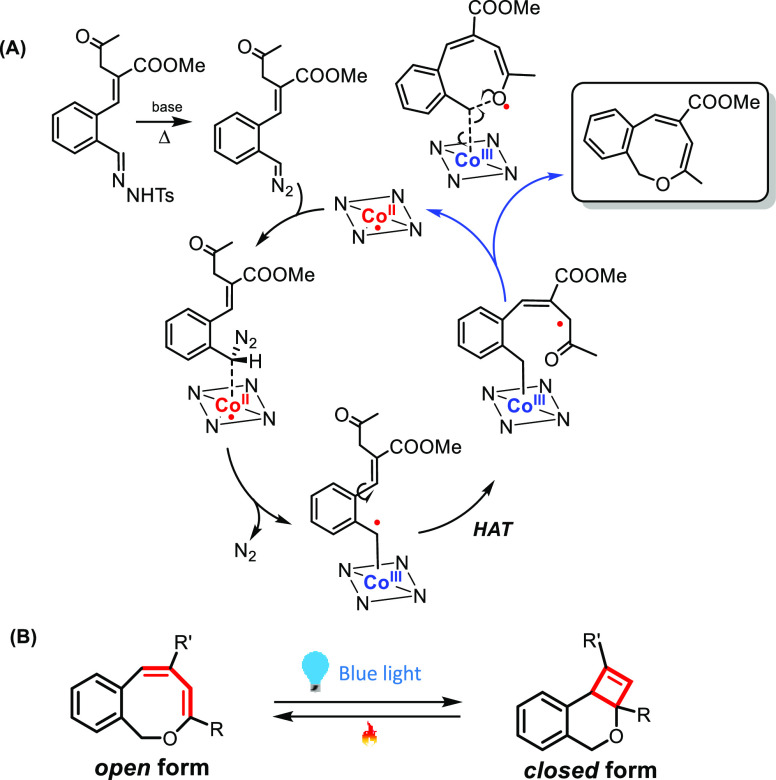
Cobalt-Catalyzed Formation of Benzoxocins and Their Application as Molecular Photoswitches (A) Catalytic cycle for the formation of benzoxocins by cobalt(II)-tetraphenylporphyrin (depicted here with the NNNN square). (B) The produced benzoxocins are new molecular photoswitches, which can be switched with blue light to the closed form and can thermally revert back to the eight-membered ring form.

Observation of benzoxocin formation is particularly noteworthy, not only because of the unexpected (net) carbene radical attack onto the ketone moiety of the substrate (Scheme 14A)^75a^ but also because these new eight-membered ring compounds act as new molecular photoswitches (Scheme 14B).^75b^

### Enantioselective Catalysts

**Figure 7:**
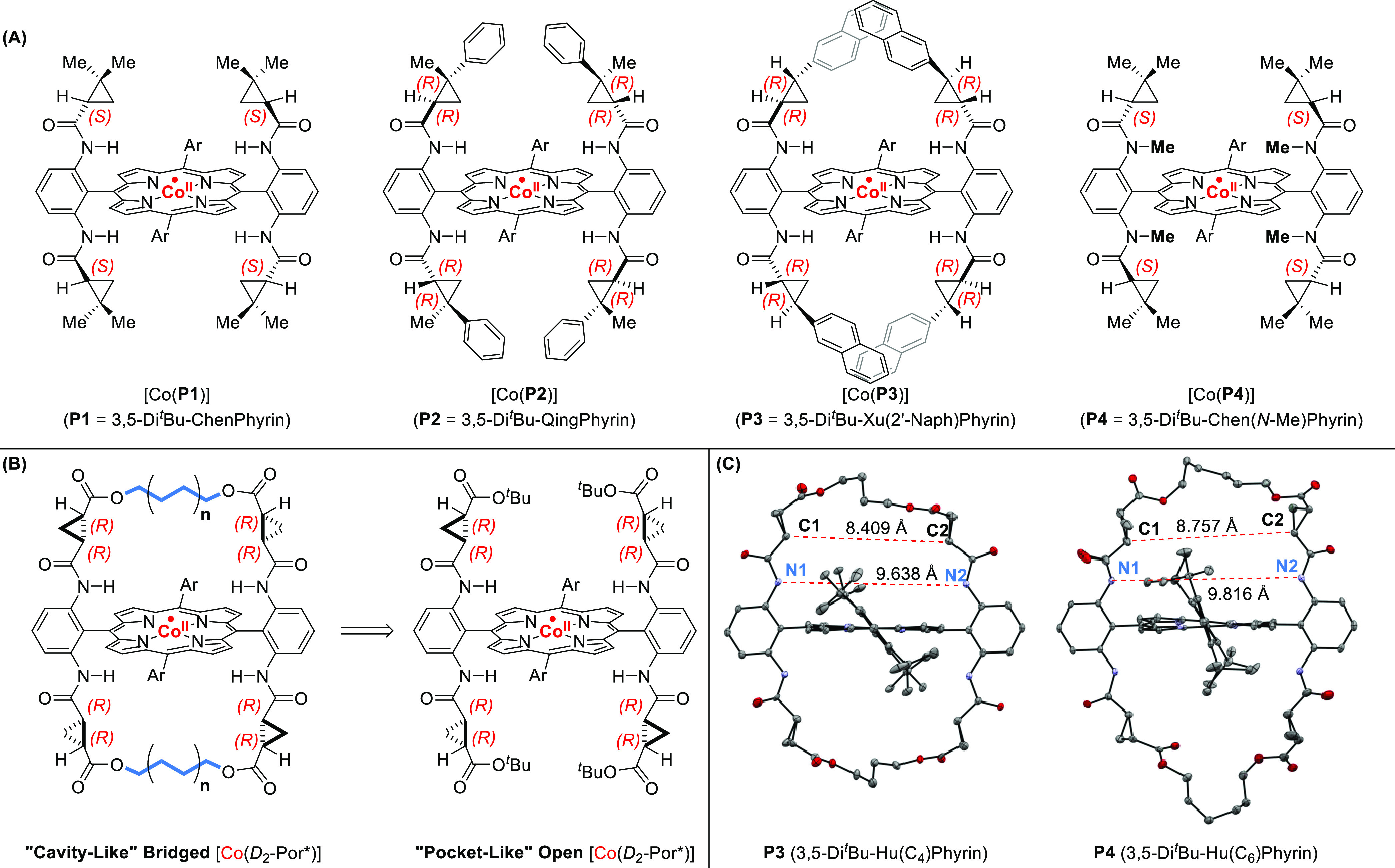
(A) Several prominent chiral hydrogen-bonding porphyrins, which were developed by the group of Zhang. (B) Next-generation chiral porphyrins with a cavity-like active site due to distal bridging of the chiral ancillary groups. (C) Crystal structures of these bridged chiral porphyrins, with the thermal ellipsoid at 50% probability.

Owing to the success of the Co(II)–porphyrin catalysts for carbene transfer reactions applied to the synthesis of many biologically and pharmaceutically relevant motifs, numerous enantioselective variants were developed by the group of Zhang.^67b,68,70,76,77^ Based on the *meso*-tetraarylporphyrin scaffold, chiral hydrogen-bonding amide groups were introduced on the *o*-aryl position (Figure 7). This bioinspired ezyme-like pocket stabilizes carbenes featuring hydrogen bond acceptors such as α-carbonyl groups. In combination with particular steric groups on other *meso*-aryl positions, these catalysts give exceedingly high chemo-, regio-, and enantioselectivity. However, much like natural enzymes, they also are quite substrate-specific. Consequently, the group of Zhang developed several variants of similar hydrogen bonding porphyrins, changing the steric bulk of the chiral groups or auxiliary *meso*-aryl moieties. The latest generation of these so-called “Zhang porphyrins” features distal bridging of the pendant chiral amide groups, which markedly increases their overall selectivity. The reduction in entropy due to the additional conformational locking of the pendant chiral groups creates an even more selective pocket for enantioselective catalysis. Interestingly, not only do these “Zhang porphyrins” exhibit excellent selectivity but they are also able to activate acceptor–acceptor diazo substrates. As such, this library of carbene radical transfer catalysts has been applied extensively toward the asymmetric synthesis of various interesting molecules, such as cyclopropanes,^76^ cyclobutanones,^65^ and indolines.^67^

**Scheme 15:**
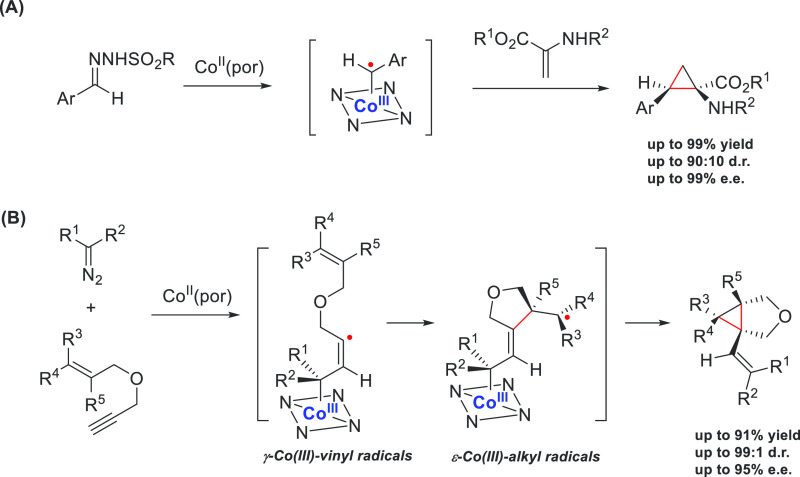
Asymmetric Synthesis Catalyzed by Chiral Cobalt Porphyrins (A) Asymmetric cyclization reactions catalyzed by chiral cobalt porphyrins and asymmetric cyclopropanation of cyclopropyl α-amino acids. (B) Radical bicyclization for cyclopropane-fused terahydrofurans.

With the assistance of cobalt(II)-based metalloradical catalysis, asymmetric cyclopropapation of dehydroaminocarboxylates with diazo compounds produces a range of functionalized α-amino-cyclopropanecarboxylates with high enantioselectivity (Scheme 15A).^76e^ Chiral cyclopropyl α-amino acids are important building blocks of peptides used in biological studies, but synthetic protocols for the direct construction of these compounds are scarce. The enantioselective radical cyclopropanation of alkenes with diazo precursors could be further applied to the synthesis of other challenging compounds. For instance, a new asymmetric strategy to construct 1-alkenylbicyclo[3.1.0]hexanes has been developed: by using 1,6-enynes and α-cyano-diazaoacetates as substrates, cyclopropane-fused terahydrofurans could be obtained in high yields with excellent enantioselectivities and diastereoselectivities (Scheme 15B).^64b^ The formation of cyclopropane-fused terahydrofurans involves a unique process of radical bicyclization: first, the γ-Co(III)-vinyl radical intermediate undergoes facile 5-*exo*-trig radical cyclization, then the generated ε-Co(III)-alkyl radical intermediate proceeds via 3-*exo*-trig cyclization, producing cyclopropane-fused terahydrofurans as products while regenerating the catalyst. It seems that the hydrogen bond interactions between the substrates and chiral catalysts are responsible for controlling the enantioselectivities of these reactions.

**Scheme 16:**
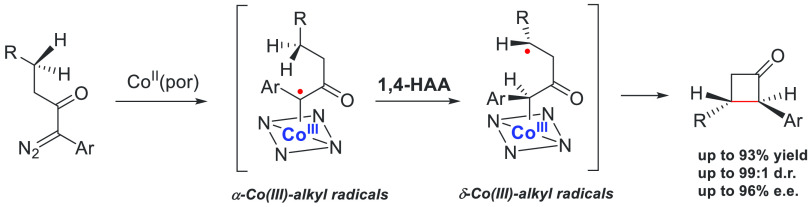
Asymmetric Construction of Cyclobutanones via Cobalt(III)–Carbene Radicals

In addition to radical addition to unsaturated bonds as a crucial step in the aforementioned transformations, hydrogen atom abstraction (HAA) is another important pathway involved in many asymmetric metalloradical catalyses. With the bridged *D*_2_-symmetric chiral amido-porphyrin as the optimal supporting ligand, the Co(II)-based metalloradical system can catalyze the asymmetric 1,4-C–H alkylation of α-aryldiazoketones, affording α,β-disubstituted cyclobutanones in good yields with high enantioselectivities (Scheme 16).^66^ The computational studies indicate that the four-membered cyclic compounds were generated through a radical mechanism that proceeds in a stepwise manner: upon metalloradical activation by the chiral cobalt catalyst, the substrate converts to an α-Co(II)–alkyl radical (carbene radical), which is followed by 1,4-HAA to produce a δ-Co(II)–alkyl radical intermediate. The latter undergoes a unique 4-*exo*-tet cyclization through intramolecular radical substitution, yielding the chiral cyclobutanone as the final product.

### Cascade Reactions

**Scheme 17:**
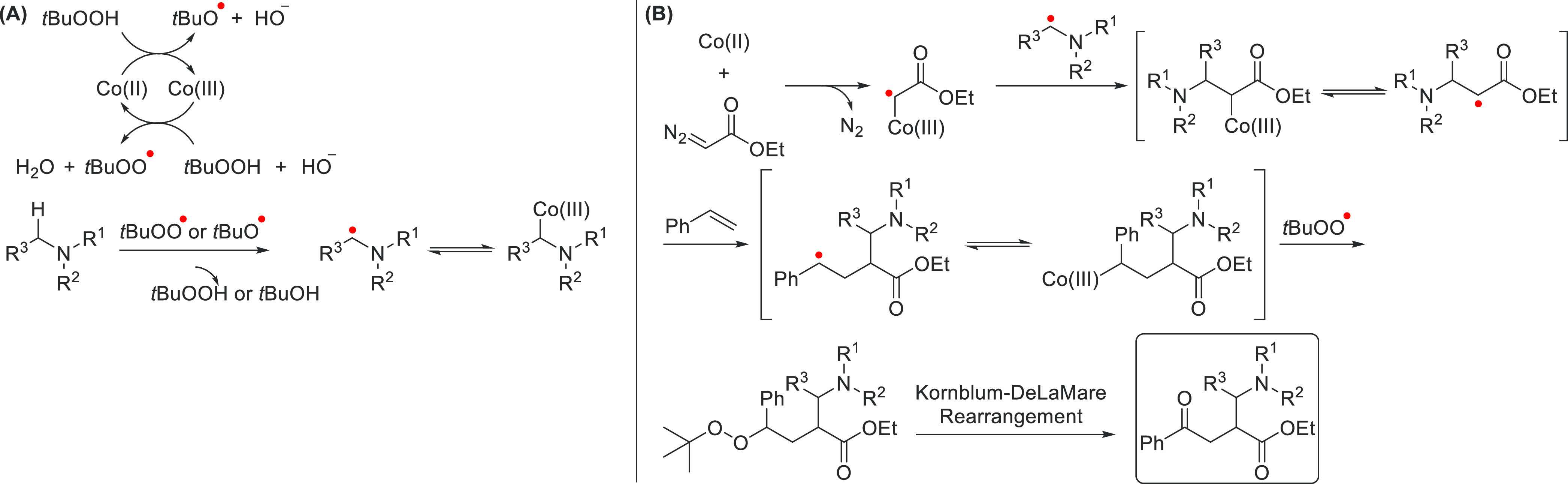
Mechanism Proposed by the Group of Wan for the Formation of β-Ester-γ-amino Ketones (A) Radicals are (re)generated by the Co^II^/Co^III^-catalyzed decomposition of *tert*-butylhydroperoxide to form reactive α-iminoradicals. (B) The carbene radical reacts with the α-iminoradical, followed by styrene and ultimately a Kornblum-DeLaMare rearrangement to form the β-ester-γ-amino ketone product.

Other examples of the application of cobalt-carbene radical species that are separate from the conventional Co(II)-mechanism shown earlier (vide supra) exist as well. One such example was reported by the group of Wan, who used a tandem reaction for the construction of β-ester-γ-aminoketones.^77^ They propose the mechanism shown in Scheme 17 based on a series of control reactions. By reacting *tert*-butylhydroperoxide with a Co(III) catalyst, the active carbene-transfer Co(II) catalyst and a peroxo radical are produced. The Co(II) catalyst reacts with ethyl diazoacetate to afford an α-ester carbene radical, whereas the peroxo radical abstracts a hydrogen from the amine to afford an α-aminoradical. The α-aminoradical proceeds to react with the carbene radical, yielding a Co(III)–alkyl adduct. This cobalt–alkyl adduct has a labile Co–C bond, which is in equilibrium with the free radical. The free radical reacts with the present arylalkene to afford a new equilibrium with the Co(II) catalyst. Another peroxoradical reacts with the benzylic carbon and via a Kornblum-DeLaMare rearrangement affords the β-ester-γ-aminoketone product.

**Scheme 18:**
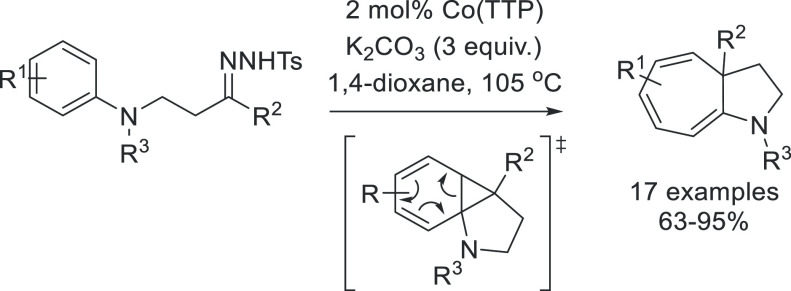
Intramolecular Buchner Reaction via a Cobalt(III)–Carbene Leading to a Range of Bicyclic Cycloheptatriene-Fused Pyrrolidines

Another instance of an alternative mechanism was an intramolecular Buchner reaction reported by Che and co-workers (Scheme 18).^64c^ An in situ generated alkyl carbene reacts with a distal aniline group at the *o*-position to afford a ring-expanded cycloheptatriene derivative. This kind of reactivity is uncommon for cobalt-based carbenes, but the high reactivity of alkyl carbenes in combination with electron-rich arenes could explain this behavior.

### Catalysis of Cobalt Phosphorus Complexes

**Scheme 19:**
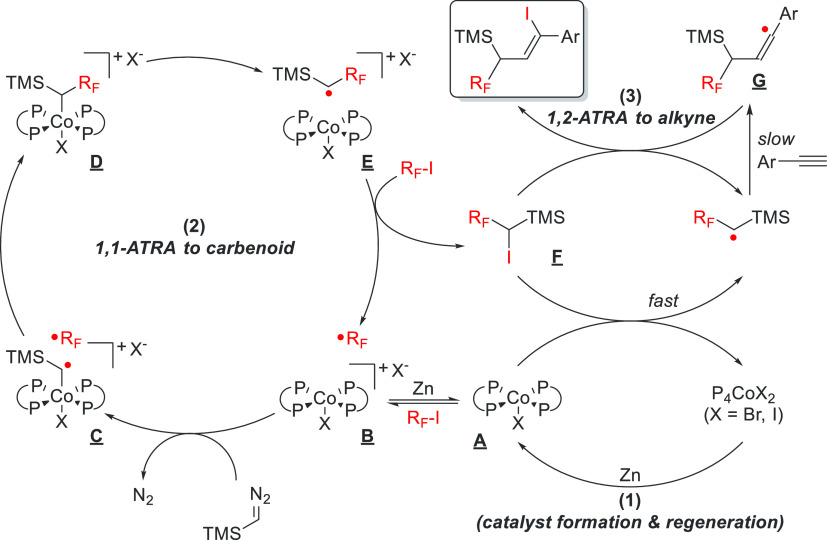
Proposed Reaction Mechanism for the Cobalt-Catalyzed ATRA Reaction by Wu et al.

Phosphorus ligands in catalysis are ubiquitous, yet not so in cobalt–carbene transfer. The group of von Wangelin used a homoleptic cobalt complex featuring two bidentate phosphine ligands to catalyze an extended atom transfer radical addition (ATRA) to synthesize highly functionalized alkenes (Scheme 19).^78^ The proposed reaction mechanism consists of multiple cycles, a main cobalt cycle and a cobalt–iodine-based secondary cycle. Reduction of a Co^II^X_2_ species to a Co^I^X species generates a nucleophilic cobalt (**A**), which is readily alkylated by the R_F_–I substrate. This cobalt–alkyl species (**B**) can undergo homolysis, freeing a Co^II^ metalloradical and an organic R_F_ radical. The Co^II^ species reacts with TMSN_2_, generating the Co^III^–carbene radical (**C**). Subsequently, the carbene radical reacts with the R_F_ radical to generate another cobalt-alkyl species (**D**), which is in equilibrium with the homolyzed metalloradical and organic free radical (**E**). Species **E** reacts with R_F_–I to form the organoiodide **F** and regenerates the active catalyst **B**. **F** can subsequently react with **A** to form the dormant Co^II^X_2_ species and a TMS(C·H)R_F_ radical, which can be trapped with an arylalkyne and another equivalent of **F** to form the final product. The mechanism is supported by radical trapping and mechanistic control experiments.

## Summary and Conclusions

As shown in this Perspective, the field of carbene radical chemistry has significantly matured in the past decade. What used to be novel insight into an organometallic peculiarity with several initial catalytic applications has now grown into a developed area of research with highly selective catalysts, giving access to a large scope of carbo- and heterocycles. Unique organic compounds can be synthesized with carbene radical catalysis, including a variety of medium sized (hetero)cyclic products with unique properties. Hydrogen-bonding cobalt-based porphyrins provide a platform for excellent enantio-, regio-, and stereoselectivity. Although catalytic application with carbene radical intermediates is largely limited to cobalt-based systems, more attention is given to using other transition metals as catalysts. Systems based on iridium, ruthenium, and copper have been reported, and significant efforts are being made to transfer this reactivity to iron. Additional insight has been gained through development of new redox-active ligand scaffolds that employ carbene radicals in their backbones. These feature a wide variety of transition metals and offer valuable insight into their unusual electronic and spectroscopic properties. Reactivity studies performed on these novel systems yield further understanding and lay the groundwork for future catalytic applications.
